# Revision of Japanese species of *Nipponomyia* Alexander, 1924 (Diptera, Pediciidae)

**DOI:** 10.3897/zookeys.1000.55021

**Published:** 2020-12-03

**Authors:** Levente-Péter Kolcsár, Daichi Kato, Maribet Gamboa, Kozo Watanabe

**Affiliations:** 1 Center for Marine Environmental Studies (CMES), Ehime University, Matsuyama, Ehime 790-8577, Japan; 2 Echigo-Matsunoyama Museum of Natural Sciences, ‘Kyororo’, 1712-2 Matsunoyama, Tôkamachi, 942-1411, Japan; 3 Department of Civil and Environmental Engineering, Ehime University, Matsuyama, Ehime 790-8577, Japan

**Keywords:** COI, crane flies, distribution, genitalia, new species, ovipositor, taxonomy, Tipuloidea

## Abstract

Japanese species of the genus *Nipponomyia* Alexander, 1924 are revised. Two new species, *Nipponomyia okinawensis* Kolcsár & Kato, **sp. nov.** and *N. yakushimensis* Kolcsár & Kato, **sp. nov.** are described from the Ryukyu Islands. Images of habitus and wings, illustrations of male and female terminalia, and distribution maps are provided for the Japanese species. A key to the world species of *Nipponomyia* is added. DNA barcodes of three Japanese *Nipponomyia* are provided, representing the first barcodes from the genus.

## Introduction

*Nipponomyia* Alexander, 1924 is a small crane fly genus belonging to the Pediciidae (Diptera: Tipuloidea). The genus was established based on the Japanese species *Tricyphona kuwanai* Alexander, 1913 and named after Japan (Nippon in Japanese). Another three species were included in the genus in the original designation, *Tricyphona novempunctata* (Senior-White, 1922) (originally described as *Amalopis*) from India, *T. symphyletes* Alexander, 1923 from Taiwan, and *T. trispinosa* Alexander, 1920 from Japan. *Nipponomyia* is morphologically characterized by the combination of the following characters: compound eye appearing bare, wing with a conspicuous yellow longitudinal stripe along the posterior margin, and gonostylus bearing 2–14 black chitinized spines (Alexander 1924, 1935, 1958). The phylogenetic position of *Nipponomyia* within the Pediciidae has not yet been investigated.

**Figure 1.**
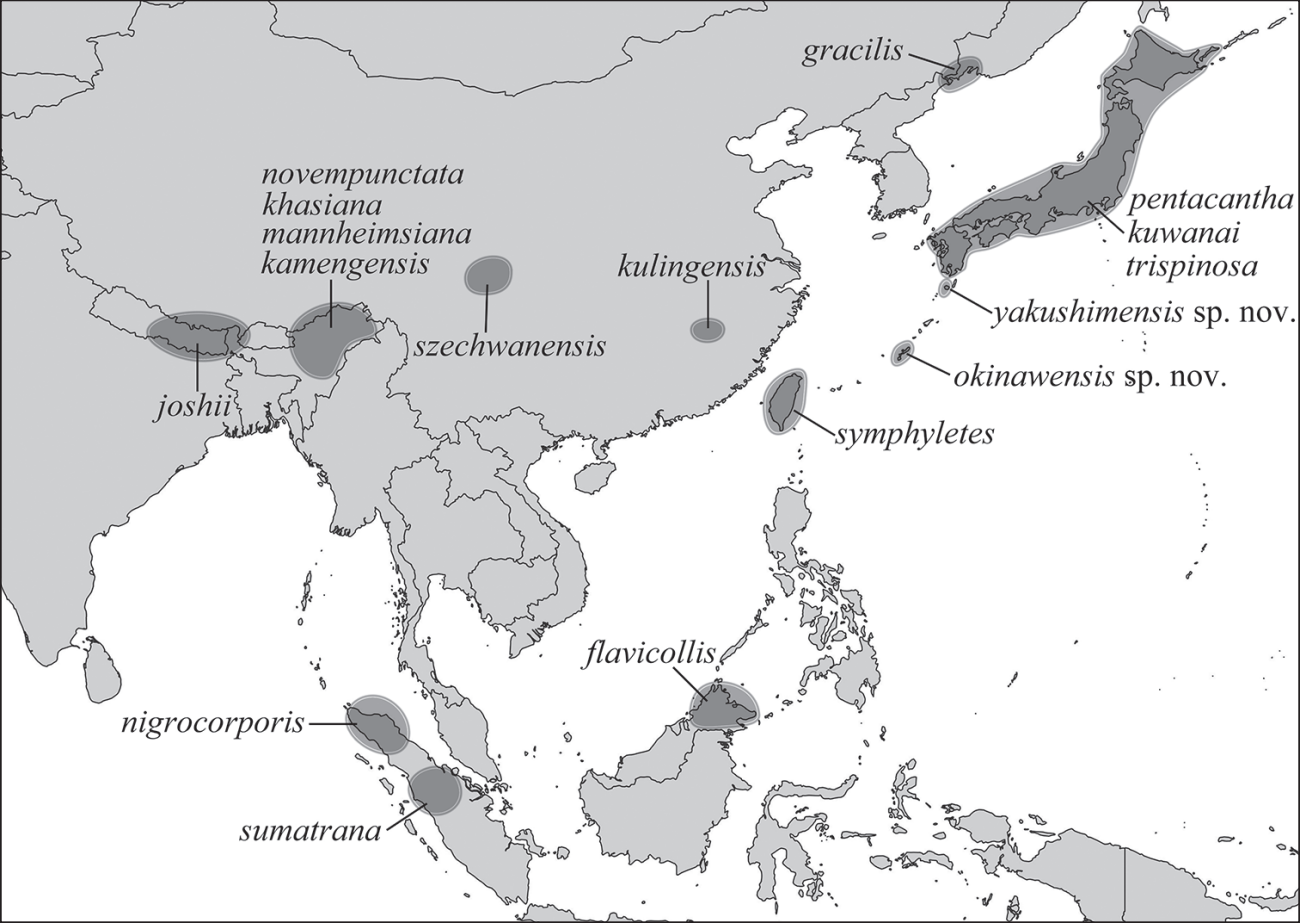
Distribution map of *Nipponomyia*.

The genus includes 15 species known from the Eastern Palearctic and Oriental regions so far (Fig. 1) (Oosterbroek 2020). The only key to the group was published by Alexander (1935), who included seven species and a partial key to the three Japanese species (Alexander 1958). The biology and immature stages of the species are unknown. The only bionomic note about *N. trispinosa* is that they swarm in the air, close to the ground at dusk (Alexander 1927a, 1958).

In the present paper we review the genus and describe two new species, *N. okinawensis* Kolcsár & Kato, sp. nov. and *N. yakushimensis* Kolcsár & Kato, sp. nov. from Ryukyu Islands, Japan. A descriptive note of the genus, images of wings and habitus, and illustrations of male and female terminalia are presented. Additional faunistic records and distribution maps are reported for Japanese species. A key to the world species of the genus is provided based on information from the literature. Finally, we present DNA barcodes for *N. kuwanai*, *N. trispinosa*, and *N. pentacantha* Alexander, 1958 with GenBank accession numbers.

## Materials and methods

Fresh materials were collected using sweep nets and stored in 90% ethanol or were dry pinned. In total, 76 specimens of *Nipponomyia* belonging to five species were examined. Male and female terminalia were described and illustrated from observations in glycerol, after maceration in 10–15% KOH and neutralization with 3% acetic acid, both at room temperature. The cleared terminalia were preserved in terminalia tubes with glycerol. Illustrations were made in Adobe Photoshop CC 2019. Photographs of wing and body were taken using a Zeiss Stemi 508 stereomicroscope equipped with a Canon Kiss M digital camera. Those of terminalia were taken using a Leica M165C stereomicroscope equipped with a Leica MC170HD camera. Stack photos were combined using Zerene Stacker software. Scanning electron microscope photos were taken with a Topcon Electron Microscope SM-200. Morphological terminology in this study follows Cumming and Wood (2017), in the case of the wing venation we follow the venation system, based on McAlpine (1981) and Merz and Haenni (2000), this system is referred to as the traditional venation system in Cumming and Wood (2017: fig. 43b), with modifications based on Starý (2008) as CuA considered as Cu here. For literature collection data an approximate spatial coordinate was selected using Google Earth Pro and distribution maps were made using QGIS3 software.

### Data resources

A specimen level dataset was made available as a Darwin Core Archive (http://ipt.pensoft.net/resource?r=nipponomyia) and is deposited in GBIF (https://doi.org/10.15468/tr5595).

### DNA isolation, amplification, and sequencing

Genomic DNA was individually extracted using DNease blood tissue kits (Qiagen GmbH, Hilden, Germany) following the manufacturer’s instructions. Extracted DNA was amplified using primers LCO-1490 and HCO-2198 (Folmer et al. 1994) on a 658 bp region of the mitochondrial cytochrome oxidase I (COI, cox1) gene, with an annealing temperature of 48 °C and 40 PCR cycles. We purified the PCR products using the QIAquick PCR Purification Kit (Qiagen GmbH, Hilden, Germany) and sequenced by Eurofins Operon (Tokyo, Japan) in both directions using the same primers as mentioned above. Forwards and reverse reads were assembled and edited using CodonCode Aligner v 3.5 (Codon Code Corporation, Dedham, USA). The sequences from *N. kuwanai*, *N. trispinosa*, and *N. pentacantha* were submitted to GenBank (accession number: MT874511–MT874514). The sequences were aligned using ClustalW (Larkin et al. 2007). We calculated the pairwise genetic distance (i.e., between species) and overall mean genetic distance with the Kimura 2-parameter model on the aligned sequences using DnaSp v5.10 (Librado and Rozas 2009).

**Depositories**

**BLKU**Biosystematic Laboratory, Kyushu University, Japan;

**CKLP** Private Collection of L.-P. Kolcsár.

## Taxonomic treatment

### Nipponomyia

Taxon classificationAnimaliaDipteraPediciidae

Alexander, 1924

D5C92A25-F65D-5116-B1B1-3813B178B06F

Figs 2 , 3 , 4 , 5 , 6 , 7 , 8 , 9 , 10 , 11 , 12 , 13 , 14 , 15 , 16 , 17 , 18 , 19

#### Type species.

*Tricyphona kuwanai* Alexander, 1913 by original designation in Alexander (1924): pages 158–159.

#### Descriptive notes on *Nipponomyia* Alexander, 1924 based on Japanese species.

General coloration yellow to black, with or without conspicuous marking on thorax. Markings of body not differing significantly between sexes.

**Figure 2.**
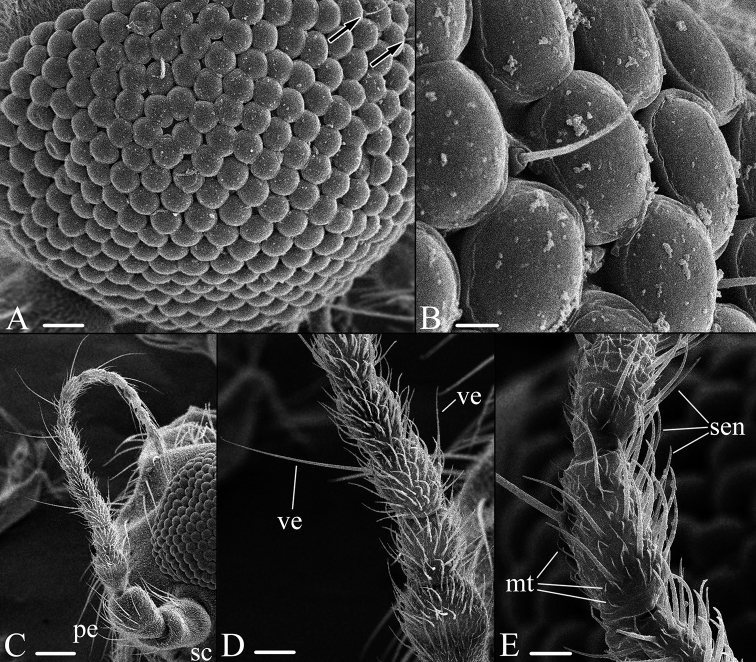
Characters of head parts of *Nipponomyia trispinosa* (Alexander), SEM **A** compound eye, 300 × (magnification) **B** compound eye, 1500 × **C** antenna, 100 × **D** flagellomeres 1 to 5, 300 × **E** flagellomere 9, 700 ×. Abbreviations: **mt** – microtrichia, **pe** – pedicel, **sc** – scape, **sen** – sensilla, **ve** – verticel. Scale bars: 33.3 μm (**A, D**), 6.67 μm (**B**), 100 μm (**C**), 14.3 μm (**E**).

***Head*:** Rostrum short. Eye appearing bare; however, a few small setae present between ommatidia, near to border of compound eye (Fig. 2A, B). Eyes widely separated. Antenna short in both sexes, only little longer than head. Scape 1.2–1.4 × longer and wider than pedicel. Pedicel 1.8–2.2 × wider than first flagellomere. Flagellum 11–13-segmented, evenly narrow toward apical segment. Flagellomeres oval to cylindrical, first 9 or 10 flagellomeres with 1 or 2 long, erected verticils dorsally (Fig. 2C, D). Last 3 or 4 flagellar segments with 3 or 4 verticils arranged irregularly. Last flagellar segment with 3 or 4 dark apical verticils, slightly curved upward, differing in shape to other verticils. Ventral part of flagellomeres densely covered with whitish sensilla, shorter than diameter of basal segment (Fig. 2E). Additional microtrichia on flagellomere (Fig. 2E). Palpi 5-segmented, length varying among species.

**Figure 3.**
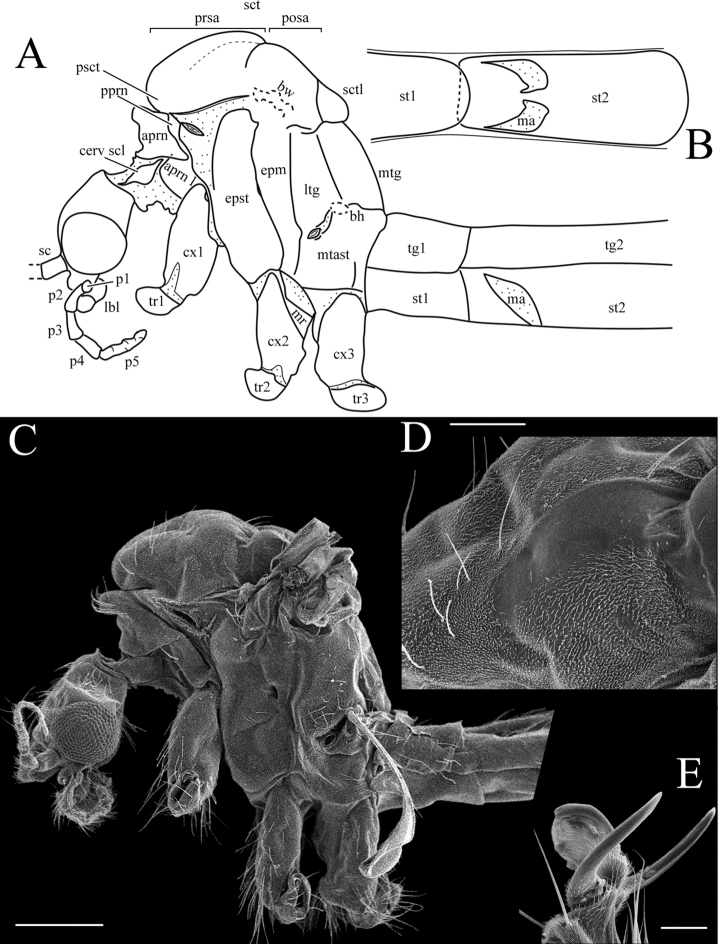
Characters of anterior body parts of *Nipponomyia trispinosa* (Alexander) **A** drawing, lateral view **B** first two sternites, ventral view **C** SEM, lateral view **D** dorsolateral view of presutural area of scutum, 150× (magnification) **E** tarsal claves 500×. Abbreviations: **aprn** – antepronotum, **bw** – base of the wing, **bh** – base of the halter, **cerv scl** – cervical sclerite, **cx** – coxa, **epm** – epimeron, **epst** – episternum, **lbl** – labellum, **ltg** – laterotergite, **mr** – meron, **ma** – membranous area of sternite 2, **mtg** – mediotergite, **mtast** – metepisternum, **p1**–**p5** – palpomeres, **pprn** – postpronotum, **prsa** – presutural area of scutum, **posa** – postsutural area of scutum, **psct** –prescutum, **scp** – scape, **sct** – scutum, **sctl** – scutellum, **st** – sternite, **tg** – tergite, **tr** – trochanter. Scale bars: 500 μm (**C**), 150 μm (**D**), 40 μm (**E**).

***Thorax*:** Elongated in dorso-ventral direction (Fig. 3A, C). Cervical sclerite elongated fusiform. Pronotum well developed, medial part of antepronotum with hump and long setae; antepronotal lobe well developed, dorsal part slipping under medial part of antepronotum; postpronotum relative narrow. Prescutum with anterior part rounded, greatly protruding anteriorly, above to the pronotum in lateral view. Scutum usually with conspicuous spots. Presutural area of scutum without longitudinal suture, just with solid line of some long hairs (Fig. 3D); area under line of hairs before transverse suture bare in SEM photo (*N. trispinosa*) (Fig. 3D); not evident under stereomicroscopes. Transverse suture deep, V-shaped, generally with dark patch in middle. Mediotergite elongated, dorsal margin almost straight in lateral view (Fig. 3A, C). Episternum, epimeron, and laterotergite each virtually not divided. Pit between episternum and epimeron deep (Fig. 3C). Meron relatively small, narrow in middle, forming two triangular parts, ventral one bigger. Metepisternum angular, additional divisions indistinct.

***Legs*:** Longer in male than in female. Fore coxa elongated, extending ventrally beyond episternum. Tibia longest segment in both sexes. Male fore tarsomere 1 as long as fore femur or slightly longer. Tibial spur formula: 1, 2, 2, spurs just half length of width of tibia. Tarsomeres with 2 spurs. Male tarsomere 5 shorter than tarsomere 4. Female tarsomere 5 longer than tarsomere 4. Tarsal claw simple, without teeth, covered with small hairs on base, arolium present (Fig. 3E). Average relative lengths of each segment (in percentage %) to the total length of corresponding leg (100%) listed in Table 1 for both sexes.

**Table 1.** The average relative lengths of each segment (as a percentage) to the total length of the corresponding leg (100%). Male data are based on *Nipponomyia kuwanai* (Alexander, 1913) (n = 8), *N. trispinosa* (Alexander, 1920) (n = 7), *N. pentacantha* Alexander, 1958 (n = 4), and *N. yakushimensis* Kolcsár & Kato, sp. nov. (n = 2), Female data are based on *Nipponomyia kuwanai* (n = 4), *N. trispinosa* (n = 6), *N. pentacantha* (n = 2), and *N. okinawensis* Kolcsár & Kato, sp. nov. (n = 1).

	Male			Female		
	Fore	Mid	Hind	Fore	Mid	Hind
**femur**	26.7	29.9	29.7	28.2	31.1	31.2
**tibia**	30.4	31.4	32.5	32.0	32.9	34.4
**tarsomere 1**	28.6	24.1	23.2	26.2	22.2	20.8
**tarsomere 2**	7.9	7.9	7.7	7.0	6.9	6.7
**tarsomere 3**	3.6	3.8	3.9	3.5	3.3	3.5
**tarsomere 4**	1.5	1.6	1.6	1.5	1.6	1.6
**tarsomere 5**	1.3	1.4	1.4	1.7	2.0	1.9

**Figure 4.**
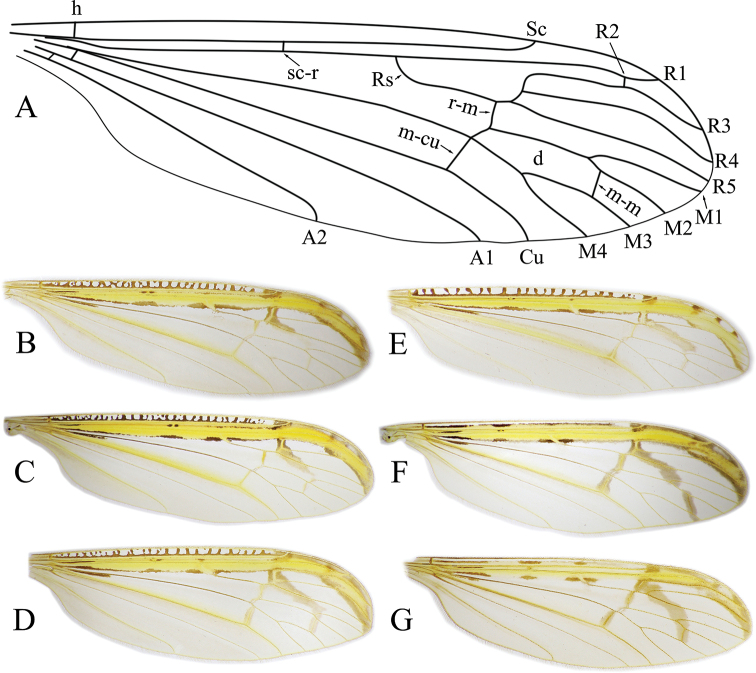
*Nipponomyia* wings **A** wing venation of *N. trispinosa* (Alexander) **B***N. kuwanai* (Alexander) from Aomori Prefecture, Honshu **C***N. kuwanai* from Ishikari Mts, Hokkaido **D***N. pentacantha* Alexander **E***N. okinawensis* Kolcsár & Kato, sp. nov. **F***N. trispinosa* (Alexander) **G***N. yakushimensis* Kolcsár & Kato, sp. nov.

***Wing*:** General wing venation as in Fig. 4A. Longitudinal veins with setae; crossveins bare. Sc long, ending beyond fork of Rs. Crossvein sc-r before origin of Rs and before or on same level as A_2_. Usually Rs forking into R_2+3+4_ and R_5_ (Fig. 4A–D, F) or rarely into R_2+3_ and R_4+5_ (Fig. 4E, G); highly variable within species. Crossvein r-m before fork of Rs, except in *N. khasiana* Alexander, 1936. R_1_ and R_3_ approaching each other at position of R_2_. Cell r_4_ wider at middle. Usually cell d closed, longer than cell m_2_. Direction of crossvein m-m variable, usually almost perpendicular (Fig. 4A–E, G) or oblique (Fig. 4F). Anterior margin of wing with yellow band, bordered with different sized and shaped brown-black patches. Additional transverse markings (dashes, dots) in costal cell present in some species (Fig. 4B–E). Additional brown markings along veins, from fork of Rs to m-m and to m-cu (Fig. 4F, G).

***Abdomen*:** Covered with relative long and dense hairs. Membranous area of second sternite well developed, shaped as in Fig. 3A, B. Usually tergites and sternites each with longitudinal dark line on lateral side (Figs 8B, 10B, 14B, 15B, 18B) and/or with spots and transverse lines (non-Japanese species).

**Figure 5.**
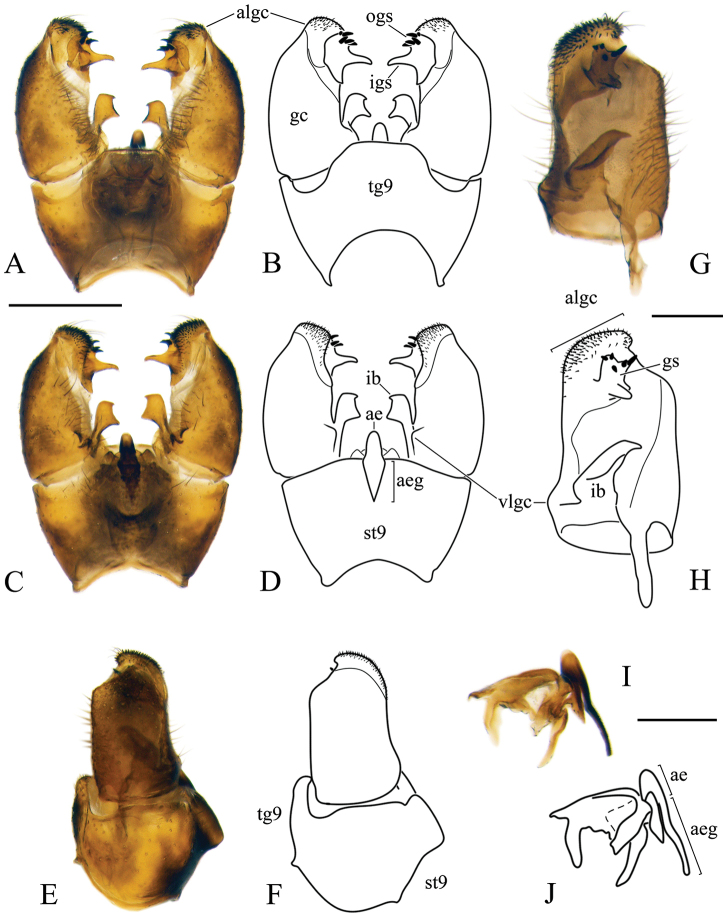
Male terminalia of *Nipponomyia pentacantha* Alexander **A, B** dorsal view **C, D** ventral view **E, F** lateral view **G, H** gonocoxite and gonostylus inner lateral view **I, J** aedeagus complex lateral view. Abbreviations: **ae** – aedeagus, **aeg** – aedeagal guide, **algc** – apical lobe of gonocoxite, **gc** – gonocoxite, **gs** – gonostylus, **ib** – interbase, **igs** – inner part of gonostylus, **ogs** – outer part of gonostylus, **st9** – sternite 9, **tg9** – tergite 9, **vlgc** – ventrobasal lobe of gonocoxite. Scale bars: 0.5 mm (**A–F**), 0.2 mm (**G, H**), 0.2 mm (**I, J**).

***Male terminalia*:** Relatively simple. Tergite 9 (epandrium) and sternite 9 (hypandrium) fused; border indistinct, forming wide ring, bulging in ventral side (Figs 5E, F, 11E, F, 16E, F, 19E, F). Tergite 9 simple without any lateral projections/arms. Gonocoxite well developed, stout, membranous on inner side. Basal lobe on ventral side of gonocoxite variable in size among species. Apical lobe of gonocoxite (sometimes referred to as outer gonostylus) partly separated from gonocoxite, elongated and directed dorso-ventrally, covered with short dark spines (Fig. 5A, B, G, H). Interbase long, well developed, fused with gonocoxite (Fig. 5G, H), with a few pale setae on ventral side. Gonostylus with two parts (Fig. 5B); inner (anterior) part of gonostylus always elongated, directed inwards; outer (posterior) part of gonostylus always shorter, wide (Figs 5G, H, 11G, H) or slender (Figs 16G, H, 19G, H) bearing 2–14 black spines. Aedeagus complex simple in shape as in most species of Pediciidae; difference among species more distinct in lateral view (Figs 5I, J, 11I, J, 16I, J, 19I, J). Aedeagus complex fused with sternite 9; relatively hard to separate from it; fused part referred in this article as aedeagal guide. Shape and length of aedeagus variable among species.

**Figure 6.**
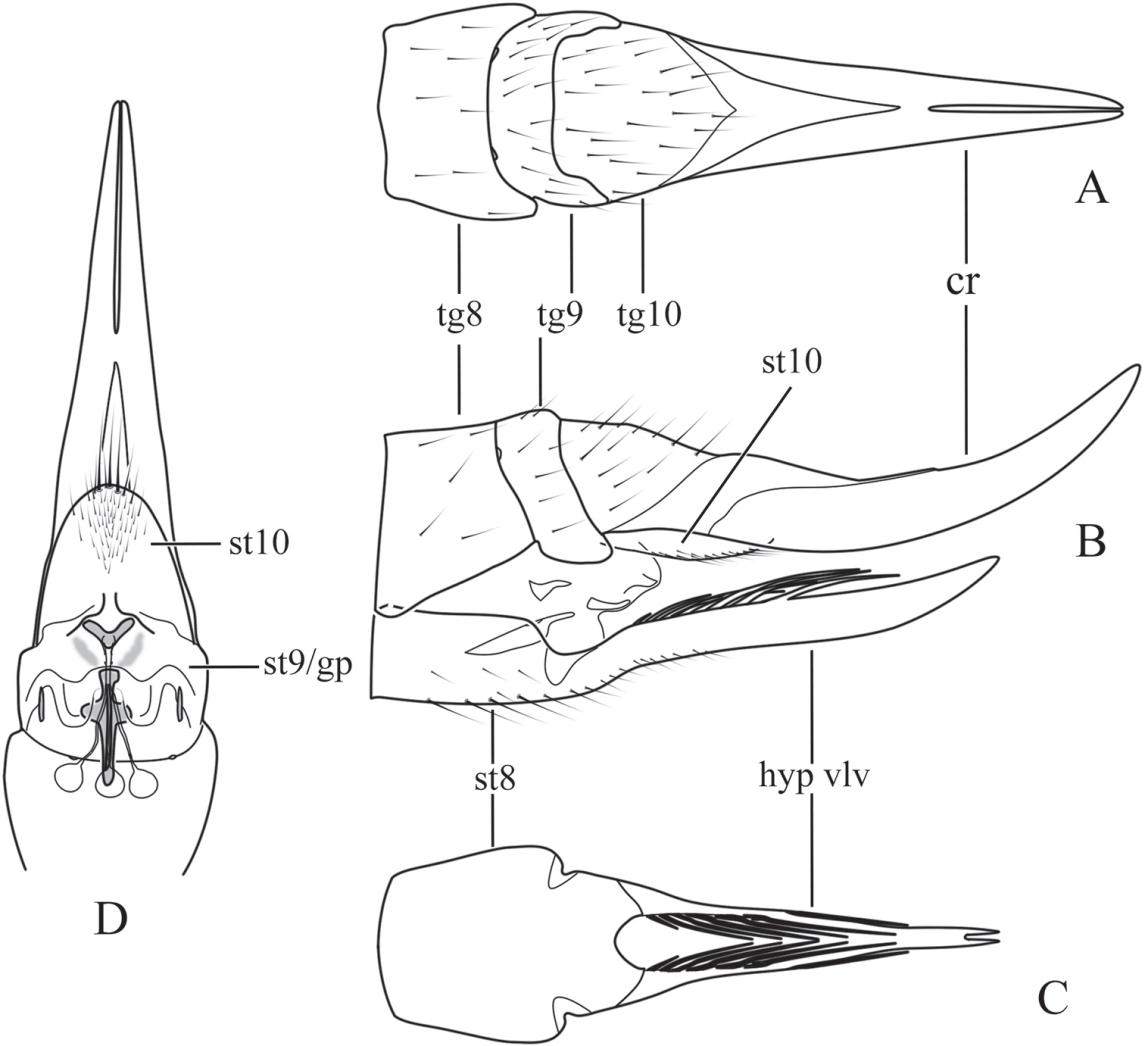
Female terminalia of *Nipponomyia pentacantha* Alexander **A** dorsal view **B** lateral view **C** inner dorsal view of sternite 8 and hypogynial valve **D** inner ventral view of sternites 9 and 10 and cerci. Abbreviations: **cr** – cercus, **hyp vlv** – hypogynial valve, **st9/gp** – sternite 9/genital plate, **st10** – sternite 10, **tg** – tergite

**Figure 7.**
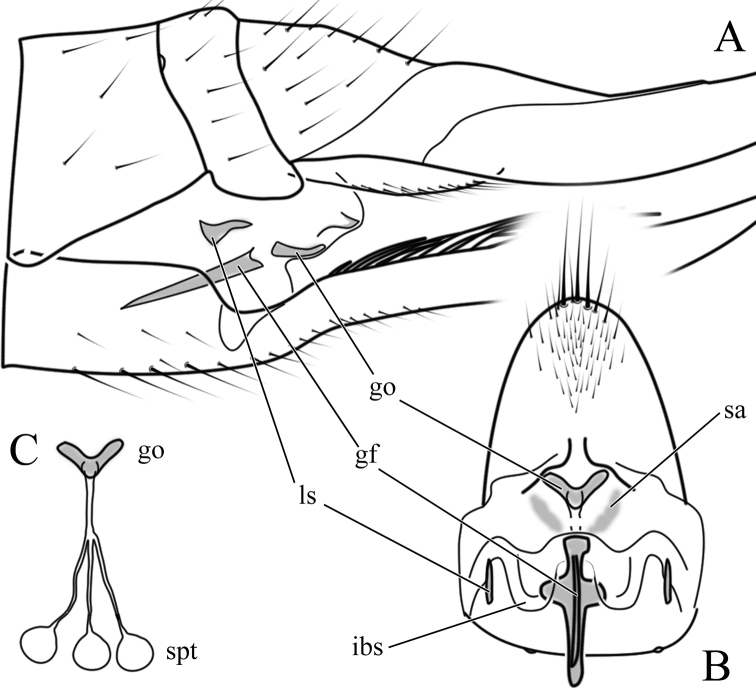
Female terminalia of *Nipponomyia pentacantha* Alexander **A** lateral view **B** sternite 9/genital plate and sternite 10, ventral view **C** genital opening and spermathecae. Abbreviations: **gf** – genital fork, **go** – genital opening, **ibs** – interbase sheath, **ls** – lateral sclerite, **sa** – sclerotized area, **spt** – spermatheca.

***Female terminalia, ovipositor*:** Elongated, tergites 8–10 fused (Fig. 6A, B). Pair of small pits situated between tergites 8 and 9. Tergite 8 at least twice as wide as tergite 9 in lateral view. Cercus longer than combined length of tergites 8–10. Cercus almost straight (Fig. 12C, E) or curving dorsally (Figs 6B, 12A). Hypogynal valve dorsally with 5–7 strong setae pointing caudally, terminal seta well separated from penultimate one and situated laterally to anterior setae (Fig. 6B, C). Genital fork well-developed, spoon-like or cruciform. Pair of membranous invaginations (“interbase sheath”) present on ventral side of genital fork, holding interbases during copulation (Fig. 7B). Sternite 9/genital plate with two sclerites lateral of genital fork, variable in shape and development among species and even within species (Figs 7A, B, 12B, D, F). Pair of sclerotized (darker) area between genital fork and genital opening present in some species. Area around genital opening sclerotized, T- or Y-shaped; Three small, light brown spermathecae closely situated to genital opening (Figs 6D, 7C). Sternite 10 rounded apically, with 5–10 longer hairs (Figs 6D, 7B, 12B, D, F).

**Larva:** Unknown.

**Pupa:** Unknown.

#### Distribution.

Eastern Palearctic and Oriental (Fig. 1).

#### Biology.

Adults swarm in the air close to the ground or above the vegetation, in shadow and windless conditions. They rest on ventral surfaces of substrates like leaves, spreading their wings horizontally, even during copulation. *Nipponomyia kuwanai* and *N. trispinosa* males walk fast on the vegetation and fly short distances to find females. *Nipponomyia kuwanai* females were observed ovipositing in muddy, wet soil, near mosses on a mountain lakeshore. A *N. trispinosa* female was observed searching for oviposition sites around wet soil, rich of organic matter next to a waterfall, but the oviposition has not yet been observed. Sometimes *N. kuwanai*, *N. trispinosa*, and *N. pentacantha* inhabit the same habitat.

### Japanese species of the genus *Nipponomyia* Alexander

**Species groups**

Japanese species of the genus can be classified into two morphological species groups. The *kuwanai* species group is characterized by the presence of black transverse lines (dashes) in costal cell (Fig. 4B–E); ultimate palpomere at most 1.6–1.7 × longer than penultimate; ventro-basal lobe of gonocoxite small, not prominent (Figs 5G, H, 11G, H); aedeagus rounded in lateral view (Figs 5I, J, 11I, J). The *trispinosa* species group is characterized by the absence of a transverse line in the costal cell (Fig. 4F, G); ultimate palpomere 1.8–3 × longer than penultimate, ventro-basal lobe of gonocoxite prominent (Figs 16G, H, 19G, H); aedeagus acute in lateral view (Figs 16I, J, 19I, J).

**Pairwise distances between species**

We successfully extracted and amplified COI barcode sequence from the three previously described species, *Nipponomyia kuwanai* (GenBank: MT874511), *N. trispinosa* (MT874512, MT874513), and *N. pentacantha* (MT874514). However, attempts to extract DNA from the type specimens of the two new species were unsuccessful. The pairwise genetic distance between species using Kimura 2-parameter ranged between 13.1% and 15.3%, the overall genetic distance is 14.2% (Table 2).

**Table 2.** Pairwise genetic distance between three *Nipponomyia* species using the COI barcoding sequences and Kimura 2-parameter.

Species	*N. pentacantha*	*N. kuwanai*	*N. trispinosa*
***N. pentacantha***	–	0.131	0.153
***N. kuwanai***	0.131	–	0.144
***N. trispinosa***	0.153	0.144	–

### Nipponomyia pentacantha

Taxon classificationAnimaliaDipteraPediciidae

Alexander, 1958

9D563E1D-CB63-5234-AC88-3B59B2DF1F92

**Figure 8.**
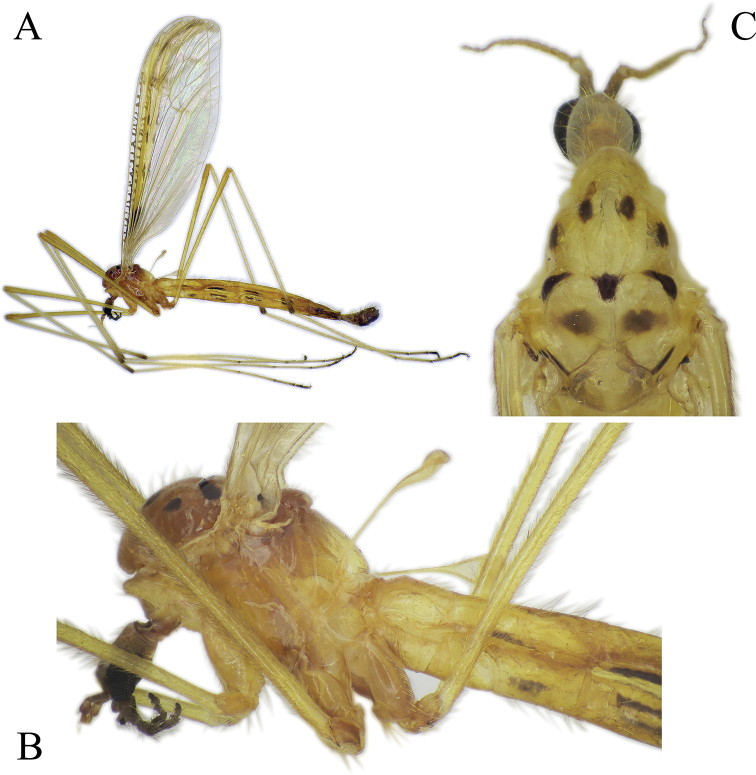
*Nipponomyia pentacantha* Alexander **A** habitus lateral view **B** anterior body parts, lateral view **C** thorax, dorsal view.

Figs 4D , 5 , 6 , 7 , 8

Nipponomyia pentacantha : Alexander, 1958: 293–294, plate 3, figs 14, 17 – original description, wing and male terminalia illustration; Ishida 1958: 39 – distribution; Nakamura 2014: 4 – distribution.

#### Type material.

***Holotype* male:** Japan, Nagano, Echigo, Mount Amakazari; alt. 300–600 m; 25–26 Jun. 1955; Baba leg. ***Paratype* male:** same location; alt. 300 m; 26 Jun. 1955; Baba leg. Type specimens deposited in National Museum of Natural History, Smithsonian Institution, Washington, D.C., USA; not studied.

#### Material examined.

**Non-types:** Japan: [Honshu] • 2♂; Aomori, Nishimeya, Shirakami Nature observation garden, Kawaratai; alt. 255 m; 40°31.13'N, 140°12.89'E; 4 Jul. 2013; leg. D. Kato (pinned, BLKU) • 1♀ (GenBank # MT874514); same data as previous except 6 Jul. 2013; D. Kato leg. (pinned, BLKU) • 1♂; Aomori, Nishimeya, Okawa Path, Kawaratai; alt. 300 m; 40°30.04'N, 140°12.24'E; 15 Jul. 2013; D. Kato leg. (pinned, BLKU) • 1♀; Aomori, Hirosaki, Inekari River, Koguriyama; alt. 170 m; 40°32.19'N, 140°29.22'E; 25 Jul. 2013; D. Kato leg. (pinned, BLKU) • 1♂; Aomori, Towada, Tsutanuma Path, Okuse; alt. 468 m; 40°35.45'N, 140°57.42'E; 21 Jun. 2014; D. Kato leg. (pinned, BLKU) • 3♂; Fukushima, Hinoemata, Hiuchigatake; alt. 1530 m; 36°59.4'N, 139°16.82'E; 16 Jul. 2019; D. Kato leg. (pinned, BLKU) • 1♂; Niigata, Tokamachi, Matsunoyama-Amamizukoshi, Mt Amamizu; alt. 920 m; 37°1.47'N, 138°33.77'E; 3 Jul. 2019; D. Kato leg. (pinned, BLKU) • 4♂, 1♀; Niigata, Tokamachi, Matsunoyama, Kyororo; alt. 310 m; 37°5.97'N, 138°36.98'E; 21 Jul. 2019; D. Kato leg. (pinned, BLKU).

#### Diagnostic characters.

Yellowish species with 11 dark spots on thorax (7 dark spots in *N. okinawensis* Kolcsár & Kato, sp. nov., 11–13 dark spots in *N. kuwanai*). Wing with transverse dark lines in costal cell. Brown marking extending from R_2+3_ to crossvein m-m (brown marking extending from R_2+3_ to maximum to base of M_1_ in *N. kuwanai* and *N. okinawensis* Kolcsár & Kato, sp. nov.). Sternite 2 without black marking at corner of membranous area (with black marking at corner of membranous area in *N. kuwanai*), a diffuse line positioned same level as line on sternite 3 (*N. kuwanai* without this line, *N. okinawensis* Kolcsár & Kato, sp. nov. with any line and dark marking on sternite 2). Gonostylus with 4 or 5 spines (11–14 spines in *N. kuwanai*). Aedeagus short, twice as long as wide, rounded (as long as wide in *N. kuwanai*). Cercus curved upward (straight in *N. okinawensis* Kolcsár & Kato, sp. nov.). Female genital opening Y-shaped (T-shaped in *N. okinawensis* Kolcsár & Kato, sp. nov.), lateral sclerite 1/3 of length of genital fork (less than 1/5 of length of genital fork in *N. kuwanai* and less than 1/6–1/7 of length of genital fork in *N. okinawensis* Kolcsár & Kato, sp. nov.), genital fork cross-shaped (spoon-shaped in *N. kuwanai*, cross-shaped in *N. okinawensis* Kolcsár & Kato, sp. nov. but lateral branch curved caudally).

#### Redescription.

***Body length*:** male 9.5–11 mm, female: 12–13 mm.

***Wing length*:** male 9.5–10.5 mm, female 10–11 mm.

***Head*:** Light brown to brown (Fig. 8C). Palpi brown, 5-segmented, palpomeres 2 to 4 subequal in length, last segment elongated, ca. 1.5 × longer than palpomere 4 in male. Female palpomere 5 almost same length as palpomere 4 or at most 1.3 × longer. Tip of palpomere 5 darker than other part of palpus. Antenna short, just a little longer than head. Scape cylindrical, wider than pedicel, twice as long as pedicel. Flagellum 13-segmented, flagellomeres gradually narrowing apically. Antenna yellow to light brown, scape always darker than pedicel and flagellomeres (Fig. 8C).

***Thorax*:** In dry specimens general coloration yellow (Fig. 8C) to fulvous (Fig. 8A, B); 4 dark spots on presutural area of scutum and 7 spots on postsutural area, sizes of spots variable, especially lateral pair of spots on presutural area (Fig. 8B, C).

***Legs*:** General coloration yellow, covered with yellowish setae (Fig. 8A). Femora without apical darkened area, apical part of tibiae brownish, with darker setae. Tarsomeres 1–3 each with narrow brown ring at tip. Tarsomeres 4 and 5 brown. Spurs on tarsomeres (2 in each segment), small but relatively easy to recognize for their darker coloration than setae.

***Wing*:** As in Fig. 4D. Wing with transverse dark lines in costal cell. Crossvein m-m present. Dark band from base of R_2+3_ extending to crossvein m-m. Dark band along crossveins r-m and m-cu pale.

***Abdomen*:** Abdomen covered with comparatively long pale setae. Tergites 2–6 in both sexes, each with longitudinal narrow black line on lateral side, situated on basal 1/3–1/2 of each tergite in male (Fig. 8A, B) and 1/2–2/3 of each tergite in females. Sternite 2 with short black line positioned on lateral side in the middle between membranous area and posterior end of sternite 2. Sternites 3–6 with a little, wider than line on tergite (Fig. 8A, B). Sometimes line on sternite 6 indistinct or absent. Tergites and sternites 7 and 8 slightly darker than previous segments, dark yellow to brown.

***Male terminalia*:** dark yellow to brown, always darkest part of abdomen (Fig. 8A). Tergite 9 almost straight at posterior margin (Fig. 5A, B). Gonocoxite without apical lobe 1.7–1.8 × longer than wide and 1.5–1.6 × longer than tergite 9 in lateral view (Fig. 5E, F). Apical lobe of gonocoxite not separated from gonocoxite, as long as 3/4 of width of gonocoxite, in lateral view (Fig. 5G, H). Posterior part of gonostylus wide, bearing 4 or 5 strong black spines (Fig. 5A, B, G, H). Interbase dilated apically, with two pointed parts; interbase with apical part 2.5–3 × as wide as basal part, in dorsal view (Fig. 5A–D). Shape of interbase in inner lateral view highly variable based on angle, directing postero-dorsally pointed at tip (Fig. 5G, H). Aedeagus short, twice as long as wide, tip rounded (Fig. 5I, J).

***Female terminalia, ovipositor*:** General coloration dark yellow. Cercus curved upward (Fig. 6B). Genital fork cross-shaped, wider in 3/4 of its length (Figs 6D, 7B). Lateral sclerite of genital plate small and narrow, 1/3 of length of genital fork (Fig. 7B). Genital opening Y-shaped, sclerotized area before genital fork relatively large, approximately as long as lateral sclerite (Fig. 7B).

**Larva:** Unknown.

**Pupa:** Unknown.

#### Distribution.

**Figure 9.**
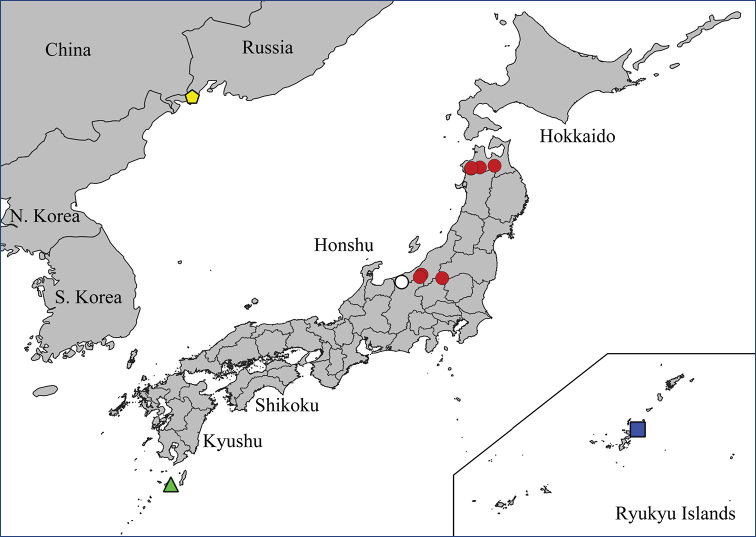
Distributions data of *Nipponomyia* species: for *N. pentacantha* Alexander white circles designate literature data while red circles are new data obtained in this study; *N. gracilis* Savchenko (yellow pentagon); *N. yakushimensis* Kolcsár & Kato, sp. nov. green triangle; *N. okinawensis* Kolcsár & Kato, sp. nov. blue square.

Japan: Honshu Island (Oosterbroek 2020, Nakamura 2014) (Fig. 9).

#### Flying period.

Middle of June to middle of September.

### Nipponomyia kuwanai

Taxon classificationAnimaliaDipteraPediciidae

(Alexander, 1913)

ADCBFAB2-6FCD-570D-97A6-E3C442AC858C

**Figure 10.**
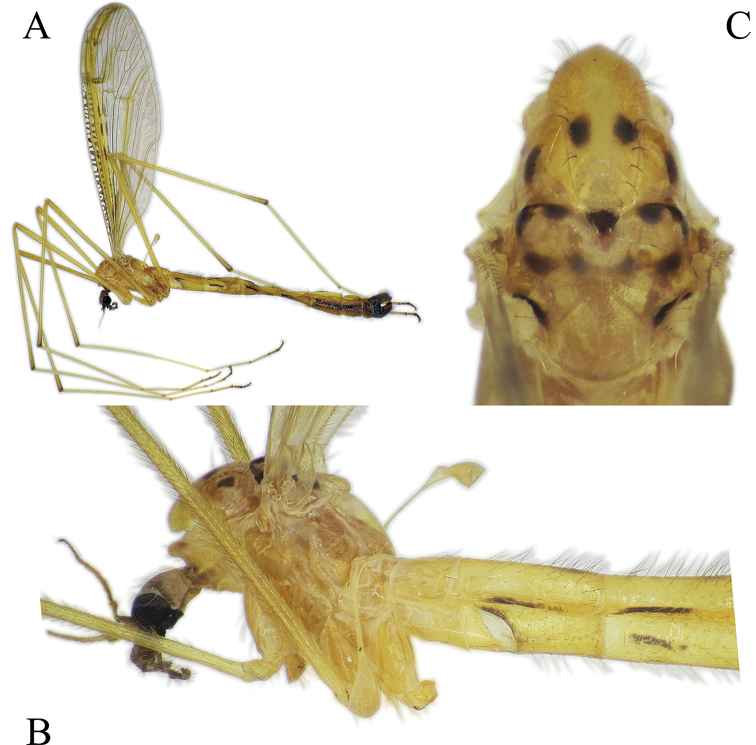
*Nipponomyia kuwanai* (Alexander) **A** habitus, lateral view **B** anterior body parts, lateral view **C** thorax, dorsal view.

**Figure 11.**
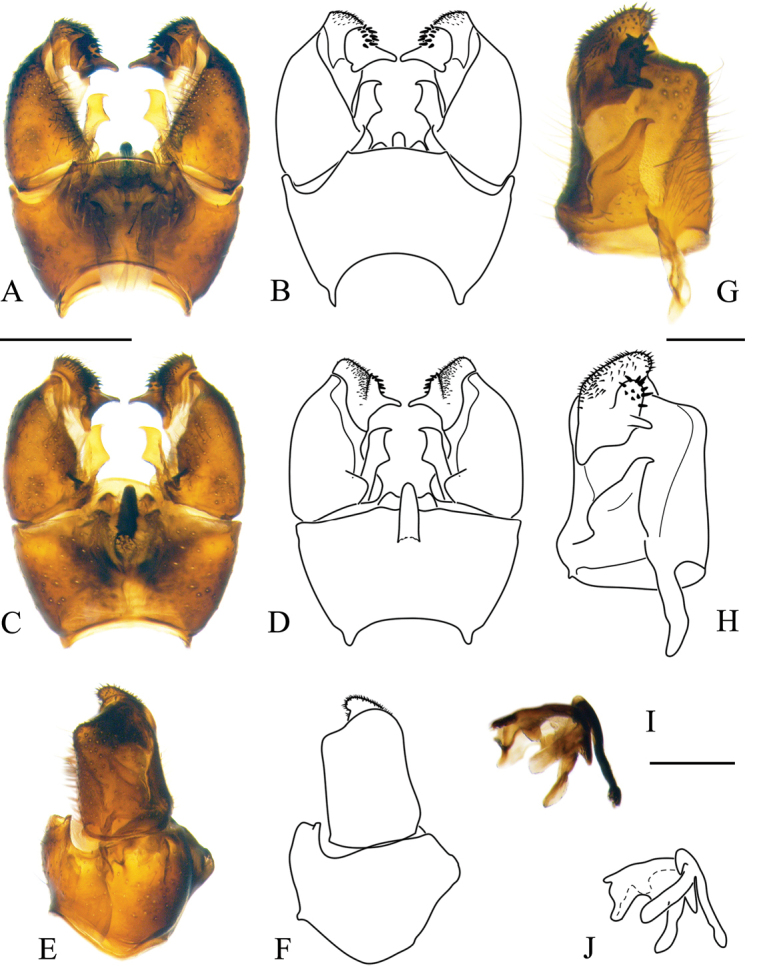
Male terminalia of *Nipponomyia kuwanai* (Alexander) **A, B** dorsal view **C, D** ventral view **E, F** lateral view **G, H** gonocoxite and gonostylus, inner lateral view **I, J** aedeagus complex, lateral view. Scale bars: 0.5 mm (**A–F**), 0.2 mm (**G, H**), 0.2 mm (**I, J**).

**Figure 12.**
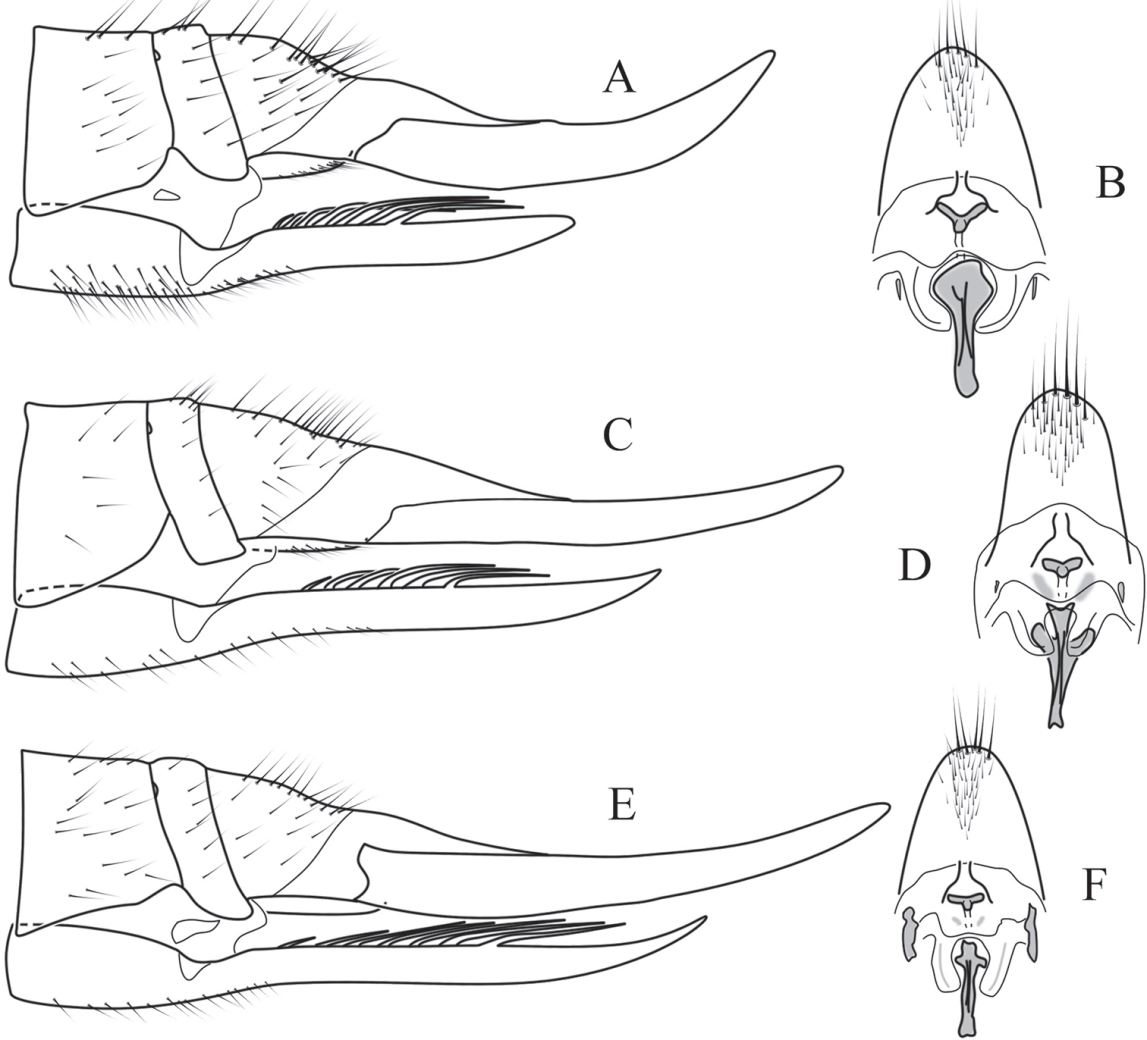
Female terminalia of *Nipponomyia* species **A, B***N. kuwanai* (Alexander) **C, D***N. okinawensis* Kolcsár & Kato, sp. nov. **E, F***N. trispinosa* (Alexander) **A, C, E** lateral view **B, D, F** ventral view of genital plate, sternite 10, and cerci.

Figs 4B, C , 10 , 11 , 12A, B

Tricyphona kuwanai : Alexander 1913: 201, 318–319, plate III, fig. 6, wing; Alexander 1920: 14–15 – male description; Alexander 1923: 479 – comparison; Alexander 1924: 158–159 – genotype of genus.
Nipponomyia kuwanai : Alexander 1927b: 49, figs 14, 15 – wing, variation, comparison; Alexander 1935: 551–552 – identification key; Esaki 1950: 1521, fig. 4363; Ishida 1958: 39 – distribution; Alexander 1958: 292–295 – identification key to Japanese species, comparison, faunistic records, Plate 3, fig. 16 male terminalia; Nakamura 2014: 4 – distribution, Japanese name; Kato and Suzuki 2017: 8 – faunistic records.

#### Type material.

***Holotype* female:** Japan, Tokyo; 7 May 1912; S.I. Kuwana leg. Type specimens deposited in National Museum of Natural History, Smithsonian Institution, Washington, D.C., USA; not examined.

***Allotype* male:** Japan, Tokyo, Meguro; 15 Apr. 1919; R. Takahashi leg. Type specimens deposited in National Museum of Natural History, Smithsonian Institution, Washington, D.C., USA; not examined.

#### Material examined.

**Non-types:** Japan: [Honshu] • 1♂; Aomori, Hirosaki, Ichinowatari-washinosu; alt. 205 m; 40°31.15'N, 140°26.33'E; 17 Jun. 2013; D. Kato leg. (pinned, BLKU) • 1♂; Aomori, Nishimeya, Shirakami Nature observation garden, Kawaratai; alt. 255 m; 40°31.13'N, 140°12.89'E; 21 Jun. 2013; D. Kato leg. (pinned, BLKU) • 1♀; Aomori, Hirosaki, Inekari River, Koguriyama; alt. 170 m; 40°32.19'N, 140°29.22'E; 26 Jun. 2013; D. Kato leg. (pinned, BLKU) • 1♂; same data as previous except 25 Jul. 2013; D. Kato leg. (pinned, BLKU) • 3♂, 2♀; Aomori, Towada, Sakura Spa, Okuse; alt. 854 m; 40°37.64'N, 140°54.59'E; 5 Aug. 2013; D. Kato leg. (pinned, BLKU) • 1♂, 1♀; Aomori, Towada, Tsutanuma Path, Okuse; alt. 468 m; 40°35.45'N, 140°57.42'E; 10 Jun. 2014; D. Kato leg. (pinned, BLKU) • 1♂; same data as previous except 21 Jun. 2014; D. Kato leg. (pinned, BLKU). [Hokkaido]: • 5♂, 1♀ (♂ GenBank # MT874511); Hokkaido, Higashikawa, Asahidake, River Yukomabetsu; alt. 1120 m; 43°39.14'N, 142°48.14'E; 23 Jul. 2019; L.-P. Kolcsár leg. (pinned or in ethanol, CKLP) • 2♀; Hokkaido, Murayama, Kijihiki Highland, muddy area; alt. 565 m; 41°57.13'N, 140°36.57'E; 30 Jul. 2019; L.-P. Kolcsár leg. (pinned or in ethanol, CKLP).

#### Diagnostic characters.

Yellowish species with 11–13 dark spots on thorax (7 dark spots in *N. okinawensis* Kolcsár & Kato, sp. nov., 11 dark spots in *N. pentacantha*). Wing with transverse dark lines in costal cell. Brown marking extending from base of R_2+3_ to base of M_1_, often not reaching M_1_ (brown marking extending from R_2+3_ to base of M_1_ in *N. okinawensis* Kolcsár & Kato, sp. nov. and to m-m in *N. pentacantha*). Second sternite with black marking at corner of membranous area (without this marking in *N. pentacantha* and *N. okinawensis* Kolcsár & Kato, sp. nov.), and without other line (a diffuse line positioned same level as line on sternite 3 in *N. pentacantha*). Gonostylus with 11–14 spines (4 or 5 spines in *N. pentacantha*). Aedeagus short, as long as wide in lateral view, tip rounded (twice as long as wide in *N. pentacantha*). Cercus curved upward (straight in *N. okinawensis* Kolcsár & Kato, sp. nov.). Genital opening Y-shaped (T-shaped in *N. okinawensis* Kolcsár & Kato, sp. nov.), lateral sclerite less than 1/5 of length of genital fork (1/3 of length of genital fork in *N. pentacantha* and less than 1/6–1/7 of length of genital fork in *N. okinawensis* Kolcsár & Kato, sp. nov.), genital fork spoon-shaped (cross-shaped in *N. pentacantha* and *N. okinawensis* Kolcsár & Kato, sp. nov.).

#### Redescription.

***Body length*:** male 9.5–11 mm, female: 12–14 mm.

***Wing length*:** male 9–12 mm, female 9.5–11.5 mm.

***Head*:** Brown with grayish pruinosity (Fig. 10B), reddish in some dry specimens, grayish pruinosity not visible in specimens stored in ethanol. Palpi brown, 5-segmented, segments 2–4 subequal in length, last segment elongated, ca. 1.5 × longer than palpomere 4 in male, maximum at most 1.3–1.4 × longer in female, measurable clearly only in specimens stored in ethanol. Tip of palpomere 5 darker than other part of palpus. Antenna short, just a little longer than head. Antenna yellow to brown, gradually lightening to apical end. Scape darker than pedicel, often color difference very contrasting. Flagellum 13-segmented, flagellomeres gradually narrowing to apical end.

***Thorax*:** General coloration yellow for specimens in alcohol, dark yellow, with reddish shade in dry specimens, dorsal parts light brown (Fig. 10). Decayed specimens more reddish; 4 spots on presutural area of scutum, lateral spots on presutural area very variable in size, and almost lacking in specimens collected in Ishikari Mountains (Asahidake, Hokkaido) and 7 or 9 spots on postsutural area of scutum. Pair of diffused spots in middle on postsutural area of scutum variable in size and shape, sometimes spots divided, forming 4 diffuse spots as in Fig. 10C.

***Legs*:** General coloration yellow, covered with yellowish setae. Femora without apical darkened area, apical part of tibia slightly brownish, with darker setae. Apical ends of tarsomeres 1–3 each with narrow brown to dark brown ring, tarsomeres 4 and 5 light brown to brown (Fig. 10A). Tarsomeres each with two spurs, small but relatively easy to recognize for their darker coloration than setae.

***Wing*:** As in Fig. 4B, C. Wing with transverse dark lines in costal cell. Dark band from R_2+3_ not extending to crossvein m-m, shorter in specimens from Honshu (Aomori prefecture) (Fig. 4B) than those from Hokkaido (Fig. 4C). Cell d closed in specimens collected by us (crossvein m-m present), open in type specimens.

***Abdomen*:** Abdomen covered with relatively long pale setae, dorsal setae darker than ventral ones. Tergites 2–6 (male) and 2–7 (female) each with a longitudinal narrow black line on lateral side, its length 1/2 of tergite in male (Fig. 10A, B) and 1/2–1 in female. Sternite 2 with short black line at corner of membranous area. Sternites 3–5, sometimes also sternite 6 with a brown line, a little wider than line on tergite (Fig. 10A, B). Sometimes line on sternite 6 less distinct or absent. Tergite and sternites 7 and 8 dark yellow to brown, darker than previous segments.

***Male terminalia*:** Dark yellow to brown (Fig. 10A). Tergite 9 with median projection almost straight at posterior margin (Fig. 11A, B). Gonocoxite without apical lobe 1.6–1.7 × longer than wide (at middle), and 1.7–1.8 × longer than tergite 9 in lateral view (Fig. 11E, F). Apical lobe of gonocoxite slightly separated from gonocoxite, more prominent in inner lateral view, as long as 2/3 of width of gonocoxite in lateral view (Fig. 11G, H). Gonostylus with 11–14 black spines, but generally with 12. Interbase dilated apically, with two pointed parts; interbase with apical part twice as wide as basal part in dorsal view (Fig. 11A, B). In inner lateral view interbase variable in shape in different angle, tip pointed and directing posterodorsally (Fig. 11G, H). Aedeagus short, as long as wide in lateral view, tip rounded (Fig. 11I, J).

***Female terminalia, ovipositor*:** General coloration dark yellow. Cercus curved upward (Fig. 12A). Genital fork spoon-shaped, wider in posterior 1/4 of its length (Fig. 12B). Lateral sclerite of genital plate, very small and narrow, less than 1/5 of length of genital fork. Genital opening Y-shaped. No chitinized area between genital fork and genital opening (Fig. 12B).

**Larva:** Unknown.

**Pupa:** Unknown.

#### Distribution.

**Figure 13:**
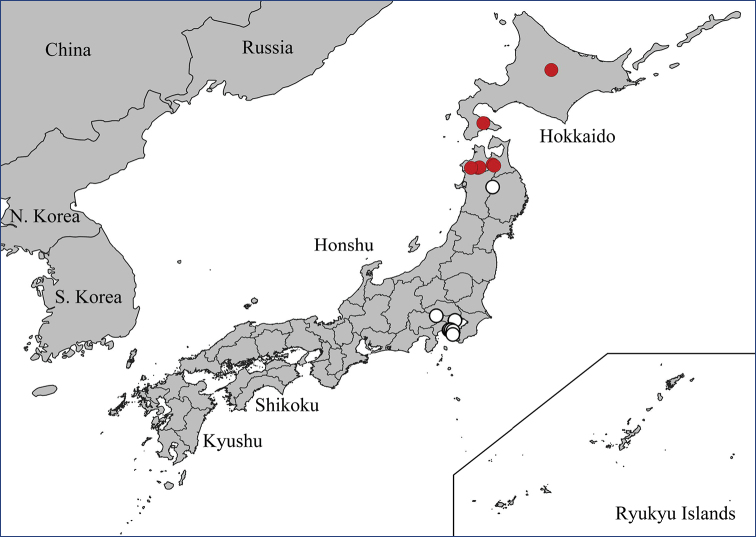
. Distribution data of *Nipponomyia kuwanai* (Alexander). White circles designate literature data, while red circles are new data obtained in this study.

Japan: Honshu (Nakamura 2014; Oosterbroek 2020), first records from Hokkaido (Fig. 13).

#### Flying period.

The species flies from April to early August.

### Nipponomyia okinawensis

Taxon classificationAnimaliaDipteraPediciidae

Kolcsár & Kato sp. nov.

2E7C04B0-F379-57B1-9F2D-4ED19A78A5E3

http://zoobank.org/E162A4C8-BE32-4590-AC19-5D2C99DEE7B4

**Figure 14.**
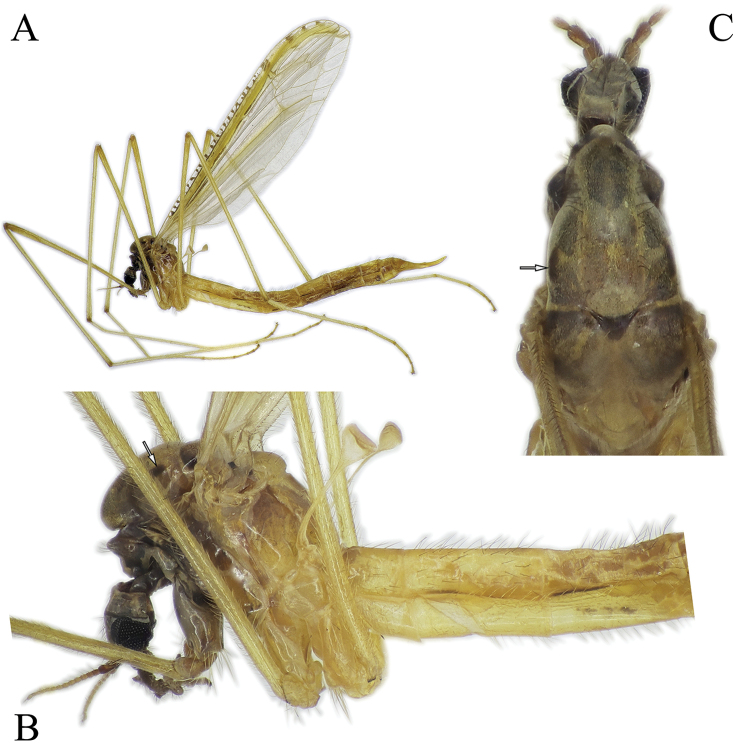
*Nipponomyia okinawensis* Kolcsár & Kato, sp. nov. **A** habitus, lateral view **B** anterior body parts, lateral view **C** thorax, dorsal view. Arrows show lateral mark on presutural area of scutum.

Figs 4E , 12C, D , 14

#### Type material.

***Holotype*** ♀, pinned. Original label: “Japan, Okinawa Island, Okinawa, Kunigami, Mt Fuenchiji-dake, Yona; alt. 250 m; 26°44.93'N, 128°14.54'E; 21 May 2016; D. Kato leg.” “***Holotype****Nipponomyia okinawensis* Kolcsár & Kato, sp. nov. [red label]” (BLKU).

#### Diagnostic characters.

Anterior part of thorax dark brown to black, posterior part yellowish brown (yellow in *N. pentacantha* and *N. kuwanai*), abdomen yellow. Thorax with 7 darker patches (11 in *N. pentacantha* and 11–13 in *N. kuwanai*), 2 in presutural area of scutum. Wing with transverse dark lines in costal cell. Brown marking extending from R_2+3_ to base of M_1_ (brown marking usually not extending to base of M_1_ in *N. kuwanai* and extending to crossvein m-m in *N. pentacantha*). Second sternite without dark line (with black marking at corner of membranous area in *N. kuwanai* and with a diffuse line positioned same level as line on sternite 3 in *N. pentacantha*). Cercus straight (curved upward in *N. kuwanai* and *N. pentacantha*). Genital opening T-shaped (Y-shaped in *N. kuwanai* and *N. pentacantha*), lateral sclerite very small, less than 1/6–1/7 of length of genital fork (1/3 of length of genital fork in *N. pentacantha* and less than 1/5 of length of genital fork in *N. kuwanai*). Genital fork cross-shaped, lateral branch curved caudally (spoon-shaped in *N. kuwanai* and cross-shaped in *N. pentacantha* but lateral branch almost straight).

#### Description.

***Body length*:** female: 12 mm.

***Wing length*:** female 10 mm.

***Head*:** General coloration brown (Fig. 14B, C). Palpus dark brown, 5-segmented, segments 2–4 almost same in length, last segment 1.5–1.6 × longer than segment 4. Tip of last flagellomere darker than other part of palpus. Antenna 1.5 × longer than head. Flagellum 13-segmented, scape and pedicel brown, flagellum gradually lightening from base to tip.

***Thorax*:** Apical half of thorax dark brown, almost black, partly due to decay inside, posterior part yellowish brown (Fig. 14). Pattern of thorax hardly recognizable, only 2 lateral large spots on presutural area of scutum distinct. Postsutural area of scutum with 5 spots, 1 triangular black spot at middle of suture, other 2 spots at anterior corners of transverse suture, and 2 small spots at posterior corners of scutum (parascutum) (Fig. 14B, C).

***Legs*:** General coloration yellow, covered with yellowish setae. Femora without apical dark area, tip of tibiae with a narrow darker ring. Apical ends of tarsomeres 1 to 4 each with narrow dark yellow to light brown ring. Tarsomeres 4 and 5 yellowish (Fig. 14A). Tarsomeres each with 2 spurs, black, easily discernible.

***Wings*:** As in Fig. 4E. Wing with transverse dark lines in costal cell. Crossvein m-m present. Narrow band on R_2+3_ not extending to crossvein m-m. Small yellowish brown area around connection of m-cu to Cu.

***Abdomen*:** Yellow to light brown, relatively short setae dark on tergites and pale on sternites. Tergites 2–6, each with longitudinal narrow black line on lateral side, 1/4–1/3 length of tergite length, less prominent compared to other species. Sternite 2 without dark mark. Sternites 3–5 each with narrow brown line, not continuous in sternite 3 (Fig. 14A, B). The abdomen removed in specimen for DNA extraction.

***Female terminalia, ovipositor*:** General coloration dark yellow (Fig. 14A). Cercus almost straight (Fig. 12C). Genital fork cross-shaped, widening at posterior 1/3, lateral branches directed caudally. Lateral sclerite of genital plate very small, indistinct, less than 1/6–1/7 of length of genital fork. Genital opening T-shaped, two darker areas between genital fork and genital opening diffuse, twice longer than lateral sclerite (Fig. 12D).

**Male:** Unknown.

**Larva:** Unknown.

**Pupa:** Unknown.

#### Distribution.

Japan: Ryukyu Islands: Okinawa Island (Fig. 9). Oriental region.

#### Flying period.

Type specimen collected at the end of May.

#### Biogeographic notes.

Okinawa Island is the largest island of the Ryukyu Archipelago, located roughly midway between Kyushu and Taiwan. The island was formed by complex process of Paleogene volcanic activities and Neogene-Quaternary sedimentations and reef deposits (Osozawa et al. 2012; Fujita et al. 2018). Okinawa is a continental island, separated and reconnected to the Eurasian mainland by land bridges few times during Neogene-Quaternary sea level fluctuations (Ota 1998). The last separation of Okinawa from mainland occurred 1.552 ± 0.154 million years ago (Osozawa et al. 2012). The island is situated in the Oriental faunal realm. The northern part of the island, the so called Yambaru Forest consists of unique, relatively well-preserved subtropical rainforest, which is home to numerous endemic plant and animal species (Ito et al. 2000). The crane fly fauna of the island very poorly known, with six species known as endemic to the island so far. The new species, *Nipponomyia okinawensis* Kolcsár & Kato, sp. nov., is most probably more closely related to the Taiwanese *N. symphyletes* than to other Japanese species; however, to support this hypothesis additional specimens must be collected from both species and both sexes.

##### Japanese species of *trispinosa* species group

### Nipponomyia trispinosa

Taxon classificationAnimaliaDipteraPediciidae

(Alexander, 1920)

5CCE30F0-2A67-56B8-8CFC-0795527F093B

**Figure 15.**
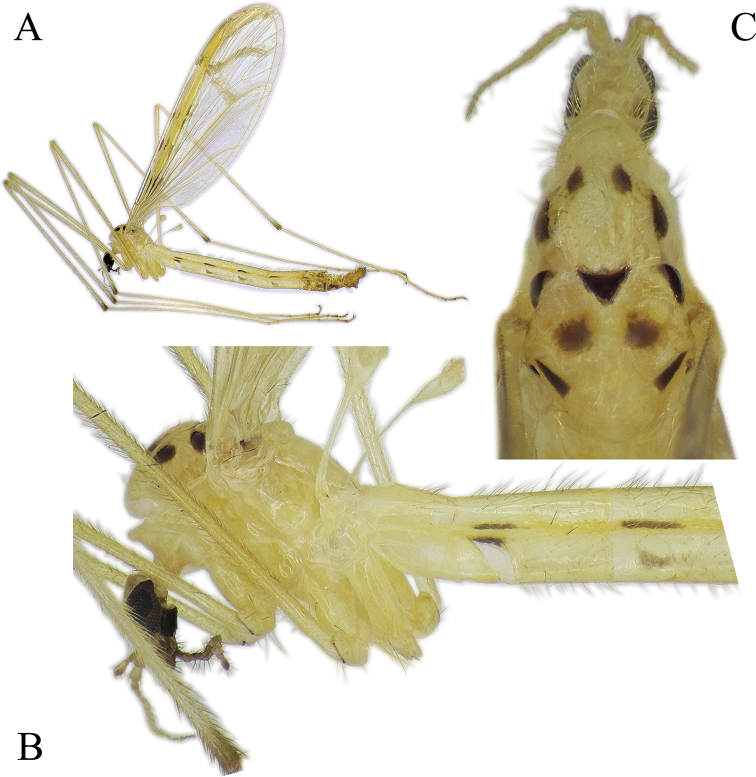
*Nipponomyia trispinosa* (Alexander) **A** habitus, lateral view **B** anterior body parts, lateral view **C** thorax, dorsal view.

**Figure 16.**
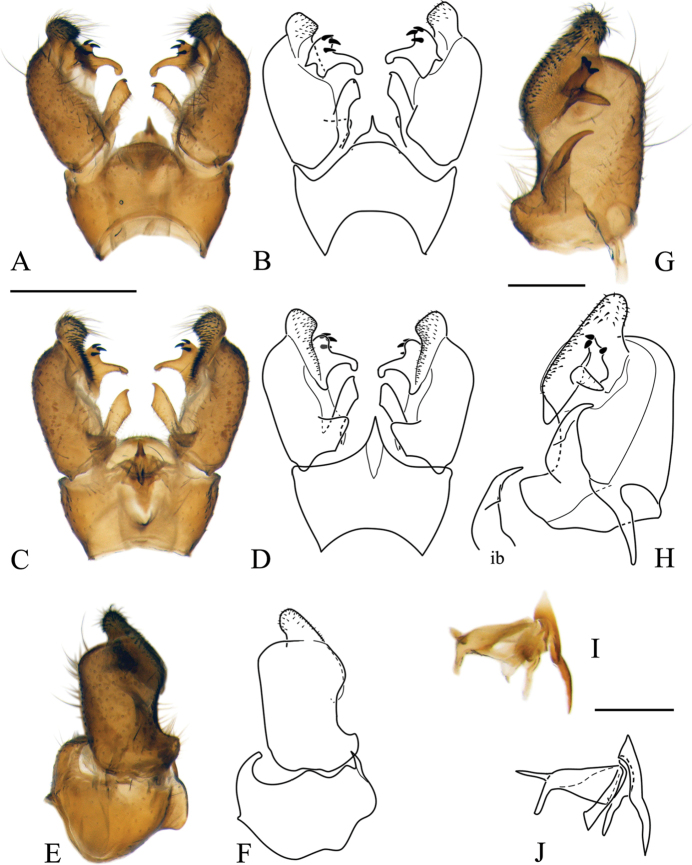
Male terminalia of *Nipponomyia trispinosa* (Alexander) **A, B** dorsal view **C, D** ventral view **E, F** lateral view **G, H** gonocoxite and gonostylus, inner lateral view **I, J** aedeagus complex, lateral view. Scale bars: 0.5 mm (**A–F**), 0.2 mm (**G, H**), 0.2 mm (**I, J**).

**Figure 17.**
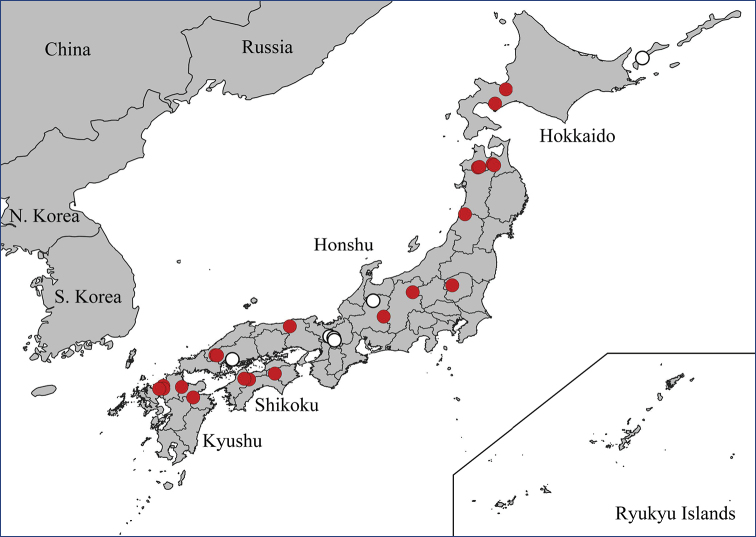
Distribution data of *Nipponomyia trispinosa* (Alexander). White circles designate literature data, while red circles are new data obtained in this study.

Figs 2 , 3 , 4A, F , 12E, F , 15 , 16 , 17

**Figure 18.**
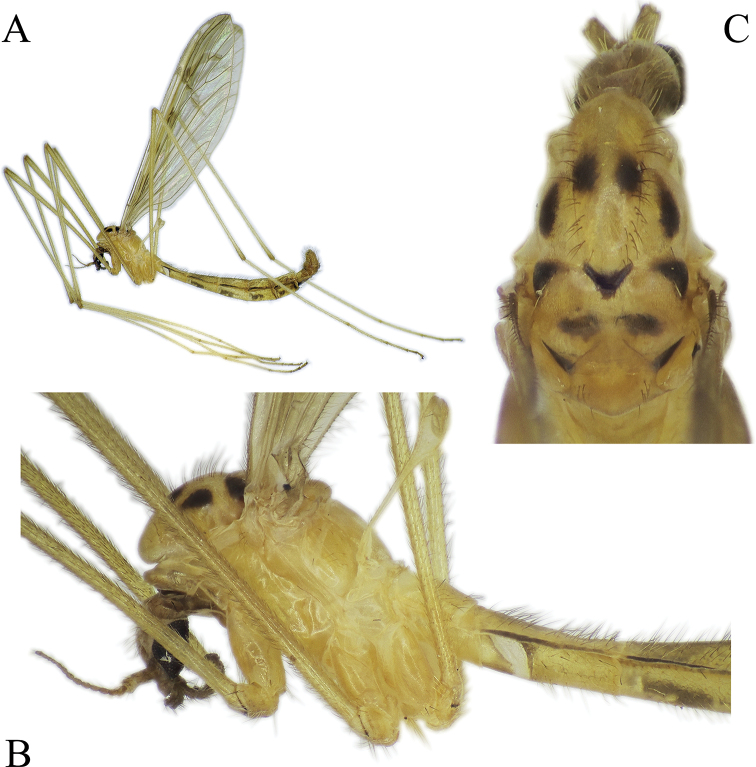
*Nipponomyia yakushimensis* Kolcsár & Kato, sp. nov. **A** habitus, lateral view **B** anterior body parts, lateral view **C** thorax, dorsal view.

Tricyphona trispinosa : Alexander 1920: 15 – original description; Alexander (1923): 479 – comparison; Alexander 1924: 158–159 – new combination to the genus.
Nipponomyia trispinosa : Alexander 1927a: 202 – faunistic record, swarming; Alexander 1927b: 49, figs 14, 15 – wing, variation, comparison; Alexander 1935: 551–552 – identification key; Alexander 1936: 190 – comparison; Esaki 1950: 1521, fig. 4362; Alexander 1958: 292–295 – identification key to Japanese species, comparison, faunistic records, Plate 3, fig. 18 – male terminalia; Ishida 1958: 39 – distribution; Savchenko and Krivolutskaya 1976: 25 – faunistic record; Savchenko 1983: 34 – comparison; Savchenko 1989 – distribution, illustration; Nakamura 2014: 4 – distribution, Japanese name.

#### Type material.

***Holotype* male:** Japan, Honshu, leg. Akio Nohiro. – without further data, probably Kyoto (see Alexander 1920, 1958). Type specimen deposited in National Museum of Natural History, Smithsonian Institution, Washington, D.C., USA; not examined.

#### Material examined.

**Non-types:** Japan: [Hokkaido] • 2♂, (GenBank # MT874512); Hokkaido, Sobetsu, River Benkei; alt. 238 m; 42°33.52'N, 140°59.29'E; 29 Jul. 2019; L.-P. Kolcsár leg. (pinned or in ethanol, CKLP). [Honshu] • 3♂, 1♀; Aomori, Hirosaki, Ichinowatari-washinosu; alt. 205 m; 40°31.15'N, 140°26.33'E; 5 Sep. 2013; D. Kato leg. (pinned, BLKU) • 1♂; Aomori, Hirosaki, Inekari River, Koguriyama; alt. 170 m; 40°32.19'N, 140°29.22'E; 10 Sep. 2013; D. Kato leg. (pinned, BLKU) • 3♂; Aomori, Towada, Tsutanuma Path, Okuse; alt. 468 m; 40°35.45'N, 140°57.42'E; 30 Aug. 2014; D. Kato leg. (pinned, BLKU) • 2♂; Aomori, Towada, Sakura Spa, Okuse; alt. 854 m; 40°37.64'N, 140°54.59'E; 3 Aug. 2015; D. Kato leg. (pinned, BLKU) • 1♂; Gifu, Nakatsugawa, Nishimata-dani Valley, Kashimo; alt. 800 m; 35°44.51'N, 137°25.57'E; 7 Aug. 2015; D. Kato leg. (pinned, BLKU) • 1♂, 1♀; Hiroshima, Hatsukaichi, Nakatsudani-gawa River, Yoshiwa; alt. 900 m; 43°0.83'N, 141°20.01'E; 2 Sep. 2015; D. Kato leg. (pinned, BLKU) • 2♂; Hiroshima, Hatsukaichi, Mt Misaka-yama, Yoshiwa; alt. 1070 m; 34°30.68'N, 132°2.81'E; 2 Sep. 2015; D. Kato leg. (pinned, BLKU) • 2♂; Nagano, Ueda, Daimyozin stream, Sugadaira MRC; alt. 1315 m; 36°31.2'N, 138°21.24'E; 20 Aug. 2013; D. Kato leg. (pinned, BLKU) • 1♂; Tochigi, Nikko; alt. 675 m; 36°44.44'N, 139°37.1'E; 8 Sep. 2011; D. Kato leg. (pinned, BLKU) • 2♂; Tottori, Yazu, Mt Ogino-sen; alt. 905 m; 35°25.85'N, 134°25.57'E; 17 Sep. 2014; D. Kato leg. (pinned, BLKU) • 1♂; Yamagata, Sakata, Yunodai Spa, Kusatsu; alt. 475 m; 39°1.57'N, 140°1.56'E; 18 Sep. 2014; D. Kato leg. (pinned, BLKU). [Kyushu] • 2♂; Fukuoka, Mt Sefuri, Itaya, Sawara-ku; alt. 970 m; 33°26.29'N, 130°22'E; 5 Sep. 2015; D. Kato leg. (pinned, BLKU) • 2♀; Fukuoka, Miyako, Notoge Pass, Saigawa-Hobashira; alt. 740 m; 33°29.74'N, 130°57.69'E; 21 Sep. 2015; D. Kato leg. (pinned, BLKU) • 1♂; Fukuoka, Fukuoka, Katae, Mt Abura; alt. 225 m; 33°31.83'N, 130°21.96'E; 20 Oct. 2015; D. Kato leg. (pinned, BLKU) • 1♀; Oita, Yufu, Shonai-cho-asono; alt. 870 m; 33°9.65'N, 131°20.21'E; 10 Sep. 2016; D. Kato leg. (pinned, BLKU) • 1♂; Saga, Saga-shi, Kasa River near Hokuza Dam, Fujimachi-sekiya; alt. 330 m; 33°25.99'N, 130°13.93'E; 15 Oct. 2015; D. Kato leg. (pinned, BLKU). [Shikoku] • 1♂; Ehime, Wakayama, small waterfall and stream; alt. 1305 m; 33°44.71'N, 133°8.23'E; 10 Sep. 2019; L.-P. Kolcsár leg. (pinned or in ethanol, CKLP) • 3♂, 1♀, (GenBank # MT874513); Ehime, Toon, Shiraino waterfall; alt. 685 m; 33°45.54'N, 132°58.16'E; 16 Sep. 2019; L.-P. Kolcsár leg. (pinned or in ethanol, CKLP) • 2♂; Tokushima, Miyoshi, Ochiai Pass, Higashiiya-Ochiai; alt. 1460 m; 33°55.33'N, 133°56.88'E; 15 May 2015; D. Kato leg. (pinned, BLKU).

#### Diagnostic characters.

Yellowish species with 11 darker spots on thorax (*N. yakushimensis* Kolcsár & Kato, sp. nov. dark yellow species with 11 large dark spots, *N. gracilis* without dark spots on thorax). Wing without transverse dark line in costal cell. Brown band running from base of R_2+3_ to tip of M_4_ and to m-cu (brown band not reaching wing margin in *N. yakushimensis* Kolcsár & Kato, sp. nov.). Dark band along crossveins r-m and m-cu conspicuous. Second sternite with black marking at corner of membranous area, but without other line. Gonostylus with 3 spines (2 spines in *N. yakushimensis* Kolcsár & Kato, sp. nov. and *N. gracilis*), aedeagus short, triangular, and acute at tip in lateral view (aedeagus long, rod-shaped in *N. yakushimensis* Kolcsár & Kato, sp. nov. and *N. gracilis*). Cercus long and straight, just gently curved upward. Genital opening T-shaped, genital fork cross-shaped, lateral sclerite large, half as long as genital fork.

#### Redescription.

***Body length*:** male 8.5–12 mm, female: 13–15 mm.

***Wing length*:** male 8.5–12 mm, female 11–12 mm.

***Head*:** Yellowish brown (Fig. 15B, C) to brown with grayish pruinosity, some dry specimens with reddish shade, grayish pruinosity not visible on specimens stored in ethanol. Palpi brown, 5-segmented, segments 2–4 subequal in length, last segment elongated, ca. 2–3 × longer than segment 4, measurable only on specimens stored in ethanol. Palpomeres 1 and 2 and tip of palpomere 5 darker. Antenna short, just a little longer than head (Figs 2C, 15C). Antenna yellow to brown, sometimes scape and pedicel darker than remainder of antenna. Flagellum unicolor or gradually lightening to apical end (Fig. 15C). Flagellum 13-segmented, flagellomeres gradually narrowing to apical end (Fig. 2C).

***Thorax*:** Specimens stored in ethanol whitish yellow. General coloration yellow, dorsal parts somewhat darker yellow in pinned specimens (Fig. 15A). Sclerites in lateral view as (Fig. 3). Four uniformly dark spots on presutural area of scutum, 7 dark spots on postsutural area of scutum (Fig. 15C).

***Legs*:** General coloration yellow, covered with yellowish setae. Femora without apical dark area, apical part of tibiae brownish, with a few darker setae. Apical ends of tarsomeres light brown. Apical half of tarsomere 4 brown, tarsomere 5 slightly lighter than tarsomere 4 (Fig. 15A). Tarsomeres with very small spurs, hardly discernable.

***Wing*:** As in Fig. 4A, F. Crossvein m-m very long, oblique, connecting close to tip of M_4_. Dark band extending from base of R_2+3_ to m-cu and to crossvein m-m, and reaching wing margin. Dark band along crossveins r-m and m-cu conspicuous.

***Abdomen*:** Abdomen covered with relatively long pale setae. Tergites 2–6 in male and 2–7 in female each with a longitudinal narrow black line on lateral side, its length ranging from 1/3–1/2 of tergite length (Fig. 15A). Sternite 2 with a short black line at corner of membranous area, but without other line (Fig. 15B). Membranous area of sternite 2 as in Fig. 3B. Sternites 3–6 each with a brown line, a little wider and shorter than tergite line (Fig. 15A). Sometimes line on sternite 6 indistinct or absent. Tergites and sternites 7 and 8 dark yellow to brown.

***Male terminalia*:** Dark yellow to brown (Fig. 15A). Tergite 9 with posterior margin rounded (Fig. 16A, B). Gonocoxite with apical lobe 1.8–1.9 × longer than wide (at middle) and around 1.7–1.8 × longer than tergite 9 in lateral view (Fig. 16E, F). Apical lobe of gonocoxite prominent in any view, as long as wide of gonocoxite at middle in lateral view (Fig. 16G, H). Ventro-basal lobe of gonocoxite prominent in any view, triangular with rounded inner peak in ventral view (Fig. 16C, D). Outer part of gonostylus slender in inner lateral view, with 3 black spines, inner part of gonostylus rod-shaped, 4–5 × longer than wide, tip curved dorsally. Interbase elongated, apical widest part 1.5 × wider than base, in dorsal view (Fig. 16A, B). Shape of interbase, directing postero-dorsally, pointed at tip (Fig. 16H). Aedeagus short, triangular and pointed in lateral view (Fig. 16I, J).

***Female terminalia, ovipositor*:** General coloration dark yellow. Cercus almost straight, only weakly curved upward (Fig. 12E). Genital fork cross-shaped, wider at posterior 1/4 of its length (Fig. 12F). Lateral sclerite of genital plate large, half as long as genital fork. Genital opening T-shaped, two darker areas between genital opening and genital fork small indistinct (Fig. 12F).

**Larva:** Unknown.

**Pupa:** Unknown.

#### Distribution.

Russia: Kuril Islands. Japan: Honshu, Shikoku (Nakamura 2014; Oosterbroek 2020), first records from Hokkaido and Kyushu (Fig. 17).

#### Flying period.

Usually flying between the end of July and the middle of November, but also collected in May.

### Nipponomyia yakushimensis

Taxon classificationAnimaliaDipteraPediciidae

Kolcsár & Kato sp. nov.

79092BEE-C9E3-5D3C-B9D0-E918F2C73F3A

http://zoobank.org/D40F45C4-F8BF-4A62-AE08-260AE606090F

Figs 4G , 18 , 19

#### Type material.

***Holotype*** ♂, pinned. Original label: “Japan, Kagoshima, Yakushima Island, Yakushima, near Shirataniunsui-kyo Valley, Yakushima-cho, alt. 600 m, 30°23.04'N, 130°34.37'E, 25 Apr. 2018, D. Kato leg.” “***Holotype****Nipponomyia yakushimensis* Kolcsár & Kato, sp. nov. [red label]” (pinned, BLKU).

***Paratype*** ♂, same data as holotype (pinned, BLKU).

#### Diagnostic characters.

Dark yellow species with 11 large darker spots on thorax (*N. trispinosa* light yellowish species with 11 smaller dark spots, *N. gracilis* brownish species without any dark spots on thorax). Wing without transverse dark line on costal cell. Brown band running from base of R_2+3_ to crossvein m-m, but not reaching wing margin (reaching the wing margin in *N. trispinosa*). Brown band along crossveins r-m and m-cu conspicuous. Second sternite with black marking at corner of membranous area, but without other line. Gonostylus with 2 spines (3 spines in *N. trispinosa*), aedeagus long, rod-shaped and acute at tip (aedeagus short, triangular in *N. trispinosa*).

#### Description.

***Body length*:** male 8–8.5 mm.

***Wing length*:** male 8–8.5 mm.

***Head*:** Light brown to brown with grayish pruinosity (Fig. 18B, C). Palpi dark brown, 5-segmented, segments 2–4 subequal in length, last segment twice as long as palpomere 4. Antenna short, just a little longer than head. Antenna brown, flagellomeres darker than scape and pedicel. Flagellum 13-segmented, flagellomeres gradually narrowing to apical end.

***Thorax*:** General coloration dark yellow, dorsal parts somewhat darker (Fig. 18A). Presutural area of scutum with 4 large spots, very conspicuous (Fig. 18B, C) and 7 spots on postsutural area of scutum also distinct (Fig. 18C). Setae on thorax relatively long and dark.

***Legs*:** General coloration yellow, covered with yellowish setae. Femora without clear apical dark area, but with some darker setae. Apical part of tibiae light brown, with a few darker setae. Apical ends of tarsomeres narrowly dark yellow to light brown (Fig. 18A). Tarsomeres with spurs very small, hardly discernible.

***Wing*:** As in Fig. 5G. Yellow pattern less intensive compared to other Japanese species. Spots around yellow costal region brown, not blackish as in *N. trispinosa*. Brown band running from base of R_2+3_ to crossvein m-m, but not reaching wing margin. Brown band along crossveins r-m and m-cu conspicuous. In paratype, wing with Rs divided to R_2+3+4_ and R_5_ (Fig. 5G), in holotype as usual in genus, divided to R_2+3_ and R_4+5_.

***Abdomen*:** Abdomen covered with relative long dark setae. Tergites 2–6 each with a longitudinal narrow black line on lateral side, its length ranging from 1/2–3/4 of tergite length. Sternite 2 with a short black line at corner of membranous fold. Sternites 3–7 each with a broad brown patch, covering anterior half of segment (Fig. 18A, B). The abdomen of paratype removed for DNA extraction.

**Figure 19.**
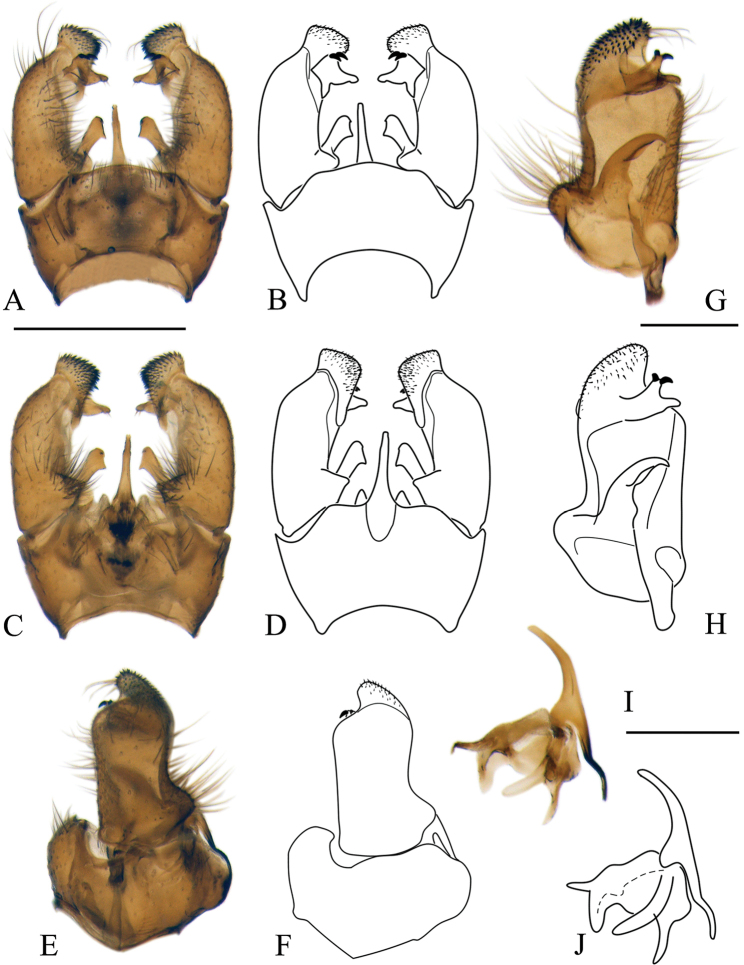
Male terminalia of *Nipponomyia yakushimensis* Kolcsár & Kato, sp. nov. **A, B** dorsal view **C, D** ventral view **E, F** lateral view **G, H** gonocoxite and gonostylus, inner lateral view **I, J** aedeagus complex, lateral view. Scale bars: 0.5 mm (**A–F**), 0.2 mm (**G, H**), 0.2 mm (**I, J**).

***Male terminalia*:** Dark yellow to light brown (Fig. 18A). Median part of tergite 9 with posterior margin convex with two small obtuse peaks laterally (Fig. 19A, B). Gonocoxite with apical lobe 2.2 × longer than wide (in the middle) and 1.6 × longer than tergite 9 (Fig. 19E, F). Apical lobe of gonocoxite squarish in dorsal and ventral views (Fig. 19A–D), as long as width of gonocoxite at middle in lateral view (Fig. 19G, H). Basal lobe of gonocoxite prominent, both in ventral and lateral views, triangular in ventral view (Fig. 19C, D). Outer part of gonostylus slender in inner view (Fig. 19G, H), with 2 black spines, inner part of gonostylus triangular (Fig. 19A–D). Interbase elongated, gradually widening to tip, widest part twice wider than basal part in dorsal view (Fig. 19A, B), interbase curved dorsally in lateral view (Fig. 19G, H). Aedeagus rod-shaped, extending beyond interbase, tip acute, curved dorsally (Fig. 19I, J).

**Female:** Unknown.

**Larva:** Unknown.

**Pupa:** Unknown.

#### Distribution.

Japan: Ryukyu Islands: Yakushima Island (Fig. 9).

#### Flying period.

Type specimens were collected at the end of April.

#### Biogeographic notes.

Yakushima Island is one of the northmost members of Ryukyu Islands, and also the largest island of the Osumi Archipelago. Yakushima is located approximately 70 km south of Kyushu and formed by a combination of sedimentary and orogenic volcanism processes (Shibasaki 2018). The island is one of the world’s wettest locations, with the annual rainfall around 10000 mm in the mountains whose peaks reach 1900 meters. The island is characterized by a unique wet climate, which ranges from subtropical to high alpine climates, and hosts numerous endemic species (Yahara et al. 1987; Smith and Kamiya 2006; Shibasaki 2018). Yakushima is located in the southern boundary of Palearctic faunal realm, and the new biogeographic boundary between the Palearctic and Oriental realm was proposed between Yakushima/Taneshima and Amami Islands (Komaki and Igawa 2017). The crane fly fauna of the island is poorly known, at the moment only six species are known as endemic to the island; however, the second author has an additional 8–10 undescribed species from Yakushima. Based on the male terminalia the new species *N. yakushimensis* Kolcsár & Kato, sp. nov. is more closely related to *N. gracilis*, than to *N. trispinosa*. Both, *N. yakushimensis* Kolcsár & Kato, sp. nov. and *N. gracilis* have 2 spines on the gonostylus, aedeagi elongated, and the shapes of their interbases are also similar. Presumably the two species diverged from each other at least 1.706 Ma ago, when the Korean Peninsula & Kyushu and also Yakushima and Kyushu separated (Osozawa et al. 2012).

### Key to world species of *Nipponomyia* Alexander

**Table:** 

1	Costal cell of wing without black transverse lines or dark points (similar to Fig. 4F, G)	**2**
–	A series of black transverse lines or points in costal cell of wing (Fig. 4B–E)	**9**
2	Presutural area of scutum with brown-black spots (Figs 15, 18)	**3**
–	Presutural area of scutum without any markings	**7**
3	Presutural area of scutum with 2 large black spots, separated by a thin yellow line, post sutural area of scutum almost black; wing with cloudy area around end of vein A_2_	***N. sumatrana* (de Meijere, 1924)**
–	Presutural area of scutum with 4 spots (similar to Figs 15, 18), wing without cloudy area around end of vein A_2_ (Fig. 4F, G)	**4**
4	Tip of the wing yellow, without darker bordering patches; only a small patch around crossvein m-cu	***N. khasiana* Alexander, 1936**
–	Tip of wing darker, yellowish pattern bordered with darker patches; distinct pattern on wing as in Fig. 4F, G	**5**
5	In addition to lateral black line on tergite, tergites with 4 brown spots on basal half; sternite with 2 lateral longitudinal lines, base of gonostylus relatively long before forking, outer part with 3 or 4 spines	***N. mannheimsiana* Alexander, 1969**
–	No additional spot on tergite; only 1 dark line on lateral side of sternite; gonostylus with basal part relatively short	**6**
6	Lateral black line on tergite shorter than half length of corresponding tergite; brown line on sternite small (Fig. 15A, B); pattern around crossvein m-m extending to tip of M_4_, m-m oblique (Fig. 4F); inner part of gonostylus long, outer part bearing 3 spines; aedeagus short, triangular (Fig. 16)	***N. trispinosa* (Alexander, 1920)**
–	Lateral black line on tergite longer than half length of corresponding tergite; line on sternite forming a broad patch (Fig. 18A, B); pattern around crossvein m-m not extending to tip of vein M_4_, m-m perpendicular (Fig. 4G); inner part of gonostylus short, outer part bearing 2 spines; aedeagus long, rod-shaped (Fig. 19)	***N. yakushimensis* Kolcsár & Kato, sp. nov.**
7	Femora and tibiae uniformly yellow; gonostylus with 3 or 4 spines	***N. szechwanensis* Alexander, 1935**
–	Femora and tibiae each with dark ring at tips; gonostylus with 2 spines	**8**
8	Presutural area of scutum brownish, wing with typical pattern of genus, similar to Fig. 4F	***N. gracilis* Savchenko, 1983**
–	Presutural area of scutum light yellow; wing with 5 or 6 darker and 5 or 6 paler spots, darker spots around base of wing, at sc-r, origin of Rs, tip of Sc, and m-cu	***N. flavicollis* Edwards, 1933**
9	Thorax, abdomen, and legs uniformly black	***N. nigrocorporis* Alexander, 1944**
–	Yellowish species, with dark markings on thorax and abdomen	**10**
10	Dark markings on costal cell spot-shaped, not forming clear transverse lines	**11**
–	Costal cell with clear transverse lines as in Fig. 4B–E	**12**
11	Scutellum brown, posterior half of mediotergite brown, tip of femora darkened	***N. kamengensis* Alexander, 1967**
–	Scutellum and mediotergite yellow, tip of femora not darkened	***N. kulingensis* Alexander, 1937**
12	Central dark spot on scutal suture absent	***N. joshii* Alexander, 1957**
–	Central dark spot on scutal suture present (Figs 8C, 10C, 14C)	**13**
13	Tip of femora darkened, a dark cloud at tip of vein A_2_, extending to vein A_1_; pattern on tip of wing brown	***N. novempunctata* (Senior-White, 1922)**
–	Femora yellow; tip of A_2_ without dark patch; tip of wing yellowish with brown border	**14**
14	Legs uniformly light yellow, segments not distinctly darkened at tip	***N. symphyletes* (Alexander, 1923)**
–	Legs light yellow, segments except femora clearly darkened at tips (Figs 8A, 10A, 14A)	**15**
15	Presutural area of scutum with a large spot on each lateral side (Fig. 14B, C); dark lateral line on tergite very narrow, less conspicuous; line on sternite also less developed, sternite 2 without any dark line (Fig. 14A, B); cercus straight (Fig. 12C)	***N* . *okinawensis* Kolcsár & Kato, sp. nov.**
–	Presutural area of scutum with 4 dark spots (Figs 8C, 10C); dark lines on tergite and sternite well developed, sternite 2 with dark line (Figs 8, 10); cercus curved upward (Figs 6B, 12A)	**16**
16	Second sternite with a relatively dark, submarginal line, no line at corner of membranous area (Fig. 8B); crossvein m-m with marking (Fig. 4D); gonostylus with 4 or 5 spines (Fig. 5); female terminalia with cross-shaped genital fork (Fig. 7B)	***N. pentacantha* Alexander, 1958**
–	Second sternite without submarginal line, but with line at corner of membranous area (Fig. 10B); crossvein m-m present or absent, without markings (Fig. 4B, C); gonostylus with 11–14 spines (Fig. 11); female terminalia with spoon-shaped genital fork (Fig. 12A)	***N. kuwanai* (Alexander, 1913)**

## Supplementary Material

XML Treatment for Nipponomyia

XML Treatment for Nipponomyia pentacantha

XML Treatment for Nipponomyia kuwanai

XML Treatment for Nipponomyia okinawensis

XML Treatment for Nipponomyia trispinosa

XML Treatment for Nipponomyia yakushimensis
